# ARHI (DIRAS3)-mediated autophagy-associated cell death enhances chemosensitivity to cisplatin in ovarian cancer cell lines and xenografts

**DOI:** 10.1038/cddis.2015.208

**Published:** 2015-08-06

**Authors:** M N Washington, G Suh, A F Orozco, M N Sutton, H Yang, Y Wang, W Mao, S Millward, A Ornelas, N Atkinson, W Liao, R C Bast, Z Lu

**Affiliations:** 1 Department of Experimental Therapeutics, University of Texas M.D. Anderson Cancer Center, Houston, TX 77030-4009, USA

## Abstract

Autophagy can sustain or kill tumor cells depending upon the context. The mechanism of autophagy-associated cell death has not been well elucidated and autophagy has enhanced or inhibited sensitivity of cancer cells to cytotoxic chemotherapy in different models. *ARHI* (*DIRAS3*), an imprinted tumor suppressor gene, is downregulated in 60% of ovarian cancers. In cell culture, re-expression of ARHI induces autophagy and ovarian cancer cell death within 72 h. In xenografts, re-expression of ARHI arrests cell growth and induces autophagy, but does not kill engrafted cancer cells. When ARHI levels are reduced after 6 weeks, dormancy is broken and xenografts grow promptly. In this study, ARHI-induced ovarian cancer cell death in culture has been found to depend upon autophagy and has been linked to G1 cell-cycle arrest, enhanced reactive oxygen species (ROS) activity, RIP1/RIP3 activation and necrosis. Re-expression of ARHI enhanced the cytotoxic effect of cisplatin in cell culture, increasing caspase-3 activation and PARP cleavage by inhibiting ERK and HER2 activity and downregulating XIAP and Bcl-2. In xenografts, treatment with cisplatin significantly slowed the outgrowth of dormant autophagic cells after reduction of ARHI, but the addition of chloroquine did not further inhibit xenograft outgrowth. Taken together, we have found that autophagy-associated cancer cell death and autophagy-enhanced sensitivity to cisplatin depend upon different mechanisms and that dormant, autophagic cancer cells are still vulnerable to cisplatin-based chemotherapy.

Autophagy has a well-defined role in cellular physiology, removing senescent organelles and catabolizing long-lived proteins.^1, 2^ Under nutrient-poor conditions, the fatty acids and amino acids produced by hydrolysis of lipids and proteins in autophagolysosomes can provide energy to sustain starving cells. Prolonged autophagy is, however, associated with caspase-independent type II programmed cell death. Although the mechanism of autophagy-associated cell death has not been adequately characterized, programmed necrosis or necroptosis has been implicated in some studies.^3, 4^

Given the ability to sustain or kill cells, the role of autophagy in cancer is complex and dependent on the context of individual studies. During oncogenesis in genetically engineered mice, reduced hemizygous expression of genes required for autophagy (BECN1, Atg4, ATG5, Atg7) can accelerate spontaneous or chemically induced tumor formation,^5, 6^ suggesting that autophagy can serve as a tumor suppressor. Other observations with established cancers suggest that autophagy can sustain metabolically challenged neoplasms, particularly in settings with inadequate vascular access.^7, 8^ Autophagy has also been shown to protect cancer cells from the lethal effects of some cytotoxic drugs.^9, 10^

Our group has found that cancer cell proliferation,^11, 12, 13^ motility,^14^ autophagy and tumor dormancy^15, 16^ can be regulated by an imprinted tumor suppressor gene, *ARHI* (*DIRAS3*), that is downregulated in 60% of ovarian cancers by multiple mechanisms,^17, 18^ associated with shortened progression-free survival.^19^ Ovarian cancer cell sublines have been developed with tet-inducible expression of ARHI. In cell culture, re-expression of ARHI induces autophagy and clonogenic ovarian cancer cell death within 72 h.^16^ In xenografts, re-expression of ARHI arrests cell growth, inhibits angiogenesis and induces autophagy, but does not kill engrafted cancer cells. When ARHI levels are reduced after 6 weeks of induction, dormancy is broken, vascularization occurs and xenografts grow promptly. Treatment of dormant xenografts with chloroquine (CQ), a functional inhibitor of autophagy, delays tumor outgrowth, suggesting that autophagy facilitates survival of poorly vascularized, nutrient-deprived ovarian cancer cells. The relevance of this model to human disease is supported by the recent observation that small deposits of dormant ovarian cancer found on the peritoneal surface at ‘second look' operations following initial surgery and chemotherapy exhibit autophagy and increased expression of ARHI in >80% of cases.^20^

Ovarian cancer develops in >22 000 women each year in the United States.^21^ Over the past four decades, the 5-year survival has increased from 37% to ∼50% with optimal cytoreductive surgery and combination chemotherapy using taxane- and platinum-based regimens,^21, 22^ but long-term survival and cure stand at ∼30% for all stages, due, in large part, to the persistence and recurrence of dormant, drug-resistant ovarian cancer cells. For the past two decades, standard chemotherapy for ovarian cancer has included a combination of a platinum compound and a taxane. Carboplatin and cisplatin are alkylating agents that bind covalently to DNA producing intra- and inter-strand crosslinks that, if not repaired, induce apoptosis and cell death.^23, 24^ Our previous studies suggest that ∼20% of primary ovarian cancers exhibit punctate immunohistochemical staining for LC3, a biomarker for autophagy that decorates autophagosome membranes, whereas >80% of cancers that have survived platinum-based chemotherapy exhibit punctate LC3.^20^ Consequently, autophagy might provide one mechanism of resistance to platinum-based therapy.

In this report, we have explored mechanism(s) by which ARHI induces autophagy-associated cell death and enhances cisplatin cytotoxicity. Cisplatin has been found to trigger apoptosis by inducing caspase-3 activation and PARP cleavage in ovarian cancer cells.^25, 26^ We hypothesized that autophagy-associated cell death and autophagy-enhanced sensitivity to cisplatin depend upon different mechanisms and that dormant, autophagic cancer cells might still be vulnerable to platinum-based chemotherapy.

## Results

### Re-expression of ARHI produces cell growth arrest and clonogenic cell death

**Figure 1:**
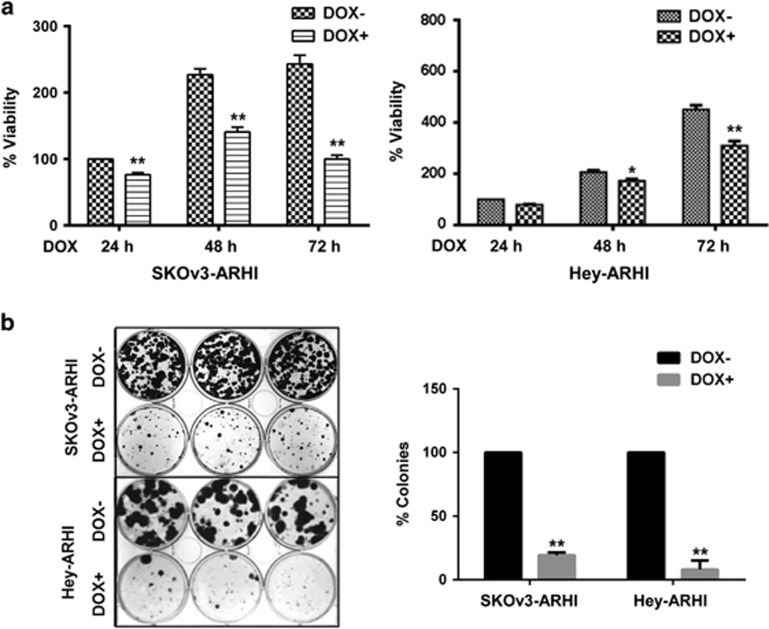
Re-expression of ARHI produced cell growth arrest and clonogenic cell death. (**a**) Re-expression of ARHI inhibited cell growth in short-term cell culture. SKOv3-ARHI cells and Hey-ARHI cells were treated with 1 *μ*g/ml DOX (to induce ARHI (DIRAS3)) for indicated time. Cell viability was measured with SRB analysis. The figure shows the combined values of three independent experiments. The columns indicate the mean, and the bars indicate the S.E. (*P<0.05; **P<0.01 DOX+ *versus* DOX−). (**b**) Re-expression of ARHI inhibited clonogenic cell growth. Cells were plated in 6-well plates, at a density of 2000 cells/well and allowed to settle for 24 h. Cells were then treated with 1 *μ*g/ml DOX for 3 days and incubated for up to 14 days. Cell viability was measured by colony counts. Data were obtained from three independent experiments. The columns indicate the mean, and the bars indicate the S.E. (**P<0.01)

ARHI expression is downregulated in the majority of ovarian cancer cell lines.^27^ In a previous report, re-expression of ARHI in cell culture induced autophagy and eliminated 90% of clonogenic SKOv3-ARHI cells within 3 days. In contrast, when ARHI was re-expressed in SKOv3-ARHI xenografts by treatment with doxycycline (DOX), autophagy was also observed, but dormant ovarian cancer cells remained fully viable for at least 6 weeks.^16^ When ARHI levels were reduced by withdrawal of DOX, xenografts grew promptly and at the same rate as noninduced controls. In the present study, we have confirmed that ARHI re-expression in cell culture decreases cell viability in a time-dependent manner in short-term sulforhodamine B (SRB) assays (Figure 1a) and that ARHI re-expression prevented clonogenic growth in both SKOv3-ARHI and Hey-ARHI cells (Figure 1b). As expected, DOX did not affect cell viability in parental SKOv3 and Hey cells (Supplementary Figure S1).

### ARHI re-expression induces G1 cell-cycle arrest, but neither apoptosis nor senescence

**Figure 2:**
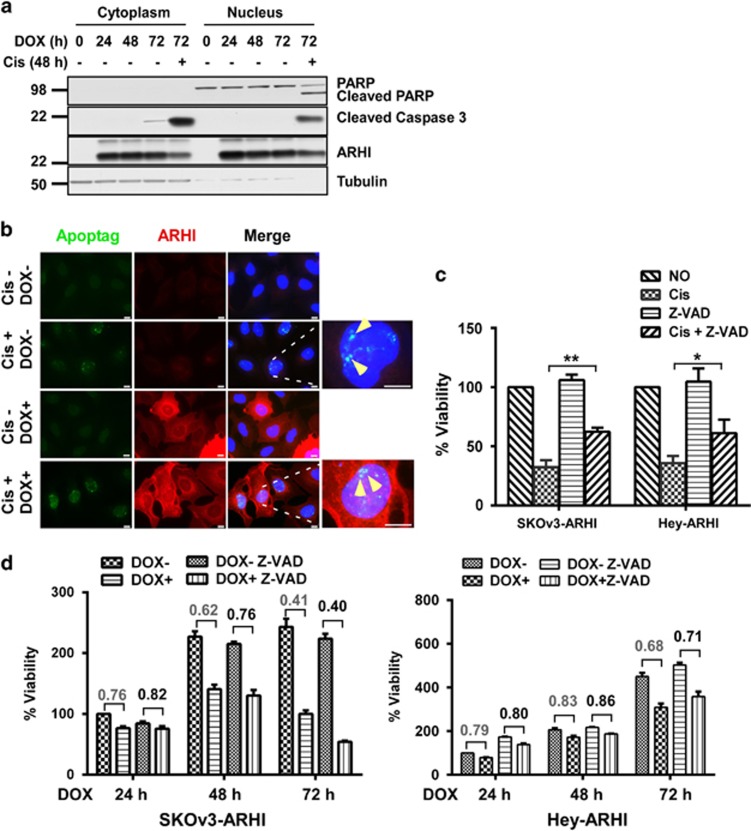
Re-expression of ARHI failed to induce apoptosis. (**a**) Re-expression of ARHI failed to induce PARP cleavage and caspase-3 activation. SKOv3-ARHI cells were treated with DOX for indicated time and one well of cells were treated with cisplatin at a final concentration of 5 *μ*M for 48 h as a positive control, and then cell lysates were collected and probed on western blots with antibodies against PARP, active caspase-3, DIRAS3 and tubulin. (**b**) Re-expression of ARHI failed to induce apoptotic cells. SKOv3-ARHI cells were treated with or without DOX to induce ARHI and with or without cisplatin (5 *μ*M) for 48 h, fixed and incubated with ARHI antibody and simultaneously with ApopTag reagent. Scale bars: 10 *μ*m. (**c** and **d**) Z-VAD inhibited cisplatin-mediated growth inhibition, but failed to block ARHI-mediated growth inhibition. SKOv3-ARHI and Hey-ARHI cell viability was measured with SRB assay as done in Figure 1a. The figure shows the combined values of three independent experiments. The columns indicate the mean, and the bars indicate the S.E. (*P<0.05; **P<0.01, cisplatin treatment *versus* no cisplatin treatment). The numbers indicate the ratio of DOX− *versus* DOX+ and ratio of DOX− Z-VAD *versus* DOX+ Z-VAD

To determine whether ARHI might inhibit growth or induce death of ovarian cancer cells by nonautophagic mechanisms, we analyzed the effect of ARHI re-expression on cell cycle, apoptosis and senescence. ARHI induction slightly, but significantly, increased the fraction of ovarian cancer cells in G1 for both SKOv3-ARHI (*P*<0.01) and HEY-ARHI (*P*<0.01) ovarian cancer cell lines (Supplementary Figure S2). A minimal increase in activated caspase-3, but no PARP cleavage, was observed after induction of ARHI (Figure 2a), whereas treatment with a low concentration of cisplatin (5 *μ*M) significantly increased activated caspase-3, and PARP cleavage, demonstrating that SKOv3-ARHI cells were capable of undergoing apoptosis (Figure 2a). Immunofluorescent staining of ARHI and ApopTag failed to detect significant apoptosis in cells that expressed ARHI in contrast to cisplatin-treated cells where ApopTag staining was readily detected (Figure 2b). Furthermore, Z-VAD, a pan-caspase inhibitor, blocked cisplatin-mediated growth inhibition, but failed to block ARHI-meditated growth inhibition (Figures 2c and d). ARHI re-expression also failed to provoke cell senescence, judged by *β*-galactosidase expression (Supplementary Figure S3). Thus, ARHI-induced growth inhibition and clonogenic cell death were associated with G1 cell-cycle arrest, but neither senescence nor apoptosis.

### Autophagy is required for ARHI-induced growth inhibition and cell death

**Figure 3:**
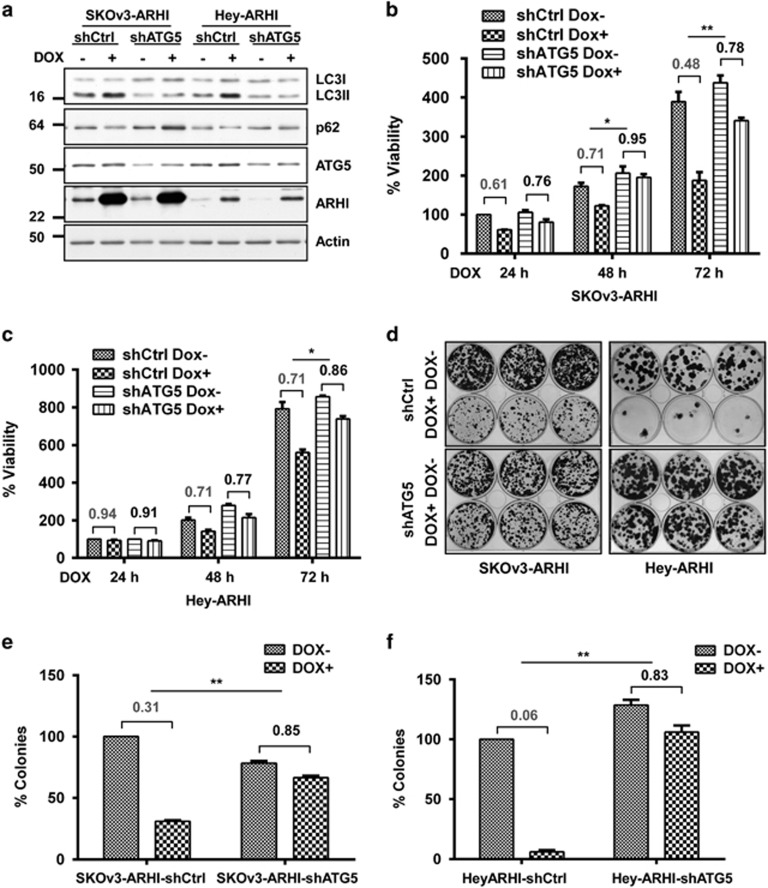
ARHI re-expression induced autophagic cell death. (**a**) ATG5 knockdown blocked ARHI-induced autophagy. SKOv3-ARHI-shControl/shATG5 cells were treated with DOX for 24 h, and then cell lysates were collected and probed with antibodies against LC3, p62, ATG5, ARHI and Actin. (**b–f**) ATG5 knockdown blocked ARHI-induced cell death. SKOv3-ARHI-shControl/shATG5 and Hey-ARHI-shControl/shATG5 cell viability was measured with SRB assays (**b** and **c**) and clonogenic assays (**d–f**) as described in Figure 1a. Each figure shows the combined values of three independent experiments. The columns indicate the mean, and the bars indicate the S.E. The numbers indicate the ratio of shCtrl DOX− *versus* shCtrl DOX+ and ratio of shATG5 DOX− *versus* shATG5 DOX+. Differences of ratio between shCtrl and shATG5 were considered statistically significant at *P<0.05 and **P<0.01

To test whether autophagy is required for ARHI-mediated growth inhibition and cell death, we established stable ATG5 and ATG7 knockdown sublines (SKOv3-ARHI-shATG5 or shATG7 and Hey-ARHI-shATG5) and control sublines that contained a scrambled shRNA (SKOv3-ARHI-shControl and HEY-ARHI-shControl). Effective knockdown of ATG5 and ATG7 protein was confirmed by western blot analysis (Figure 3a and Supplementary Figure S4A). Knockdown of ATG5 and ATG7 protein profoundly impaired the conversion of LC3 I to LC3 II and degradation of p62 after induction of ARHI re-expression with DOX, indicating inhibition of autophagosome formation (Figure 3a). Importantly, ATG5 and ATG7 knockdown essentially eliminated ARHI-mediated growth inhibition and loss of clonogenicity in SKOv3-ARHI and HEY-ARHI cells measured in both short- (Figures 3b and c and Supplementary Figure S4B) and long-term assays (Figures 3d–f and Supplementary Figures S4C and D), suggesting that autophagy is required for ARHI-induced growth inhibition and cell death.

### Re-expression of ARHI induces necrosis and upregulates ROS dependent upon autophagy

**Figure 4:**
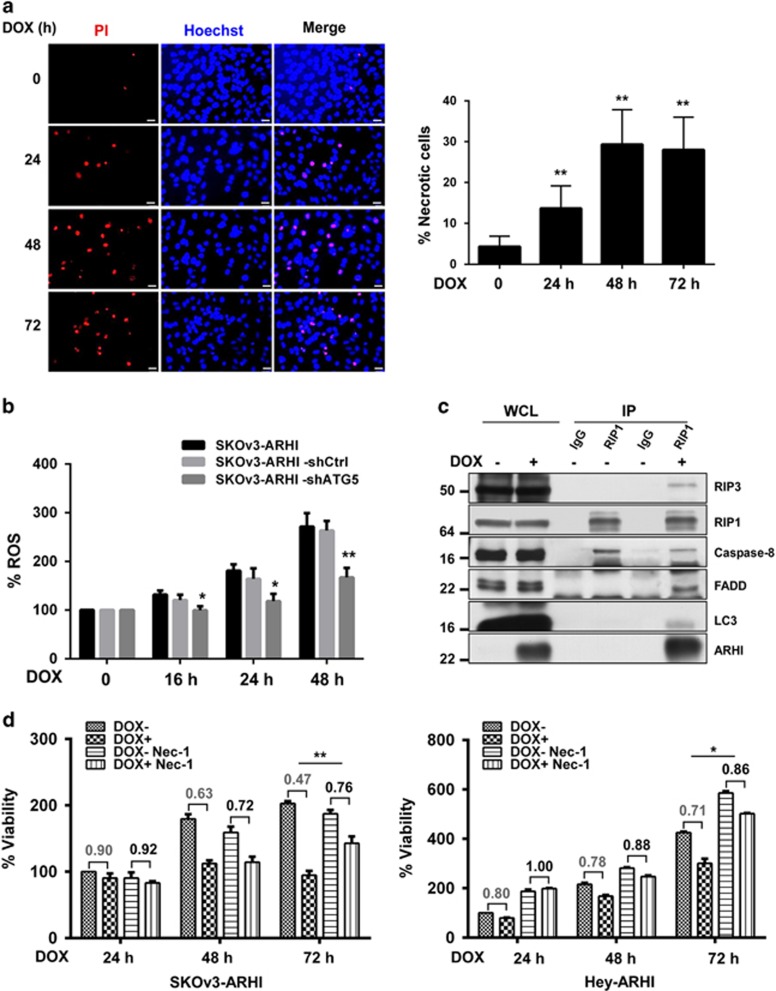
ARHI re-expression induced necrosis and reactive oxygen species (ROS) production as well as RIP1-mediated necroptosis in ovarian cancer cells. (**a**) ARHI-induced autophagy induces necrosis in ovarian cancer cells. SKOv3-ARHI cells were treated with DOX to induce ARHI expression at the intervals indicated. Live cell staining was performed with a combination of Hoechst 33342 BF (blue) and PI (red) dyes to determine the nuclear morphology and membrane integrity judged by immunofluorescence microscopy. Scale bars: 10 *μ*m. The columns indicate the mean, and the bars indicate the S.E. (**P<0.01; DOX+ *versus* DOX−). (**b**) Re-expression of ARHI induced ROS that depended upon autophagy. SKOv3-ARHI, SKOv3-ARHI-shControl and shATG5 cells were treated with DOX to induce ARHI expression at the intervals indicated and cell lysates and supernatant were collected for ROS detection. The figure shows the combined values of two independent experiments. The columns indicate the mean, and the bars indicate the S.E. (**P<0.01; shATG5 *versus* shCtrl). (**c**) ARHI interacts with RIP1 and RIP3. To examine the interaction with RIP1 and RIP3, SKOv3-ARHI cells were treated with or without DOX. Endogenous RIP1/RIP3/FADD/caspase-8/ARHI/LC3 complexes were immunoprecipitated with anti-RIP1 antibody and analyzed for co-immunoprecipitation of RIP1/RIP3/FADD/caspase-8/ARHI/LC3 conjugates (IP). Host species-matched nonspecific IgG served as negative controls. Whole-cell lysates (WCLs) are included for comparison. (**d**) Necrostatin-1 (Nec-1) significantly rescued ARHI-induced loss of cell viability. SKOv3-ARHI and Hey-ARHI cells were treated with DOX and Nec-1 (40 *μ*M) simultaneously at indicated times. Cell viability was measured with SRB assay. The figure shows the combined values of three independent experiments. The columns indicate the mean, and the bars indicate the S.E. The numbers indicate the ratio of DOX− *versus* DOX+ and ratio of DOX− Nec-1 *versus* DOX+ Nec-1. Differences of ratio between Nec-1 treatment and no treatment were considered statistically significant at *P<0.05 and **P<0.01

Using Hoechst 33342 (BF)/propidium iodide (PI) double staining, we found that ARHI re-expression induced necrosis in ovarian cancer cells. BF, a bisbenzimidazole blue fluorescence dye, penetrates intact plasma membranes and stains DNA in viable cells, whereas uptake of PI requires membrane damage and marks necrotic cells. In the absence of DOX, SKOv3-ARHI ovarian cancer cells exhibited round dark blue nuclei stained with BF, whereas SKOv3-ARHI cells treated with DOX had dark blue and red nuclei of normal size, indicating uptake of both dyes by necrotic cells (Figure 4a). Little necrosis was observed in SKOv3-ARHI-shATG5 cells when compared with SKOv3-ARHI-shControl cells (Supplementary Figure S5). One factor that can contribute to the induction of necrosis is the excessive production of reactive oxygen species (ROS) that leads to oxidative stress and damage of intracellular organelles.^28, 29^ To determine whether re-expression of ARHI increases ROS production, SKOv3-ARHI ovarian cancer cells were treated with DOX; ROS were then measured in cell lysates using Cell Biolabs OXiSelect ROS kit (San Diego, CA, USA). ARHI re-expression enhanced ROS production in a time-dependent manner (Figure 4b). Less ROS activity was generated in SKOv3-ARHI-shATG5 cells that were incapable of producing mature autophagosomes than in SKOv3-ARHI-shControl cells that underwent autophagy after treatment with DOX, suggesting that autophagy was required for ARHI-induced ROS production (Figure 4b). Furthermore, treatment with *N*-acetyl cysteine (NAC), an ROS scavenger, rescued SKOv3-ARHI ovarian cancer cells from necrosis after ARHI induction (Supplementary Figure S6), suggesting that ROS play an important role in ARHI-induced necrosis. Thus, our data suggest that re-expression of ARHI induces ROS-mediated necrotic cell death.

### ARHI-induced autophagy triggers RIP1/RIP3-mediated necroptosis

Having established that autophagy is required for ARHI-mediated necrosis in ovarian cancer cells, we next explored whether autophagy could trigger necroptosis mediated by a necrosomal complex that contained RIP1 and RIP3. An antibody against RIP1 coprecipitated RIP3, FADD and, remarkably, also ARHI and LC3 that are known to localize to the autophagosomes (Figure 4c). Inversely, an antibody against ARHI could also be coprecipitated with RIP, FADD and LC3 (Supplementary Figure S7A). ARHI-mediated autophagosome and necrosome formation was further confirmed by immunofluorescence staining in which ARHI localized with LC3 and RIP3 (Supplementary Figure S7B). Necrostatin-1 (Nec-1), a RIP1-specific inhibitor, has been reported to block RIP1/RIP3-mediated necroptosis.^30, 31^ Treatment with Nec-1 significantly decreased the ARHI-induced loss of cell viability in SKOv3-ARHI and HEY-ARHI ovarian cancer cells after treatment with DOX (Figure 4d and Supplementary Figure S7C) and reduced the induction of necrosis after ARHI re-expression (Supplementary Figure S8). In addition, RIP3 alone and RIP1 plus RIP3 knockdown decreased ARHI-mediated growth inhibition in SKOv3-ARH cells (Supplementary Figure S9). Taken together, ARHI re-expression stimulates the assembling of autophagosome–necrosome co-complexes and triggers RIP1/RIP3-mediated necrotic cell death.

### ARHI re-expression enhances chemosensitivity to cisplatin in cultured ovarian cancer cells

**Figure 5:**
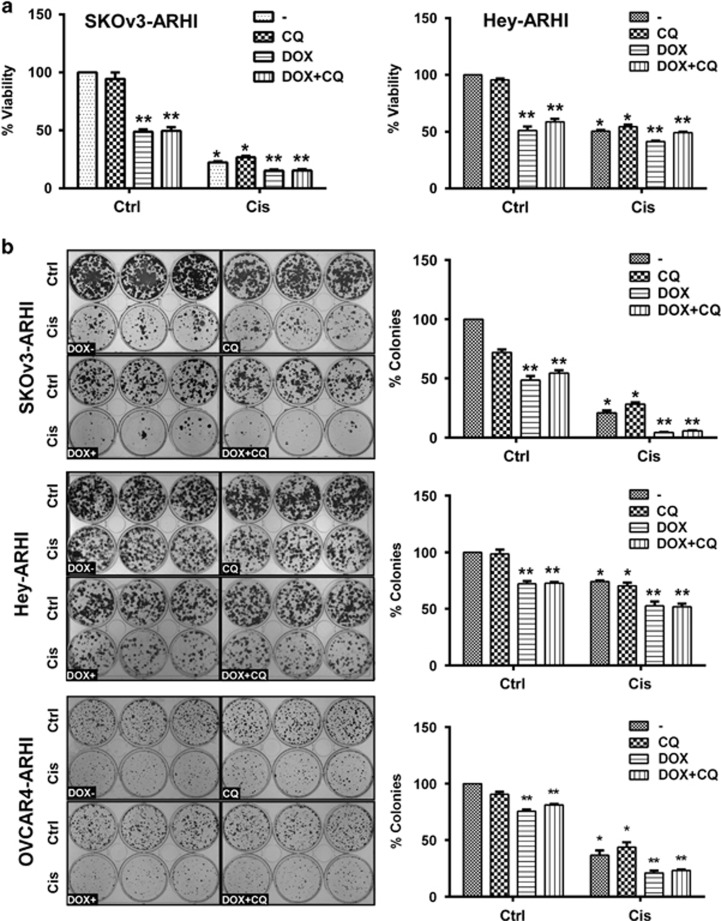
ARHI re-expression enhanced chemosensitivity to cisplatin *in vitro.* (**a**) ARHI enhanced cytotoxicity to cisplatin in short-term cell cultures. SKOv3-ARHI and Hey-ARHI cells were pre-treated with 5 *μ*M chloroquine and 1 *μ*g/ml DOX for 24 h. Cells were then treated with 5 *μ*M chloroquine (CQ), 1 *μ*g/ml DOX and 5 *μ*M cisplatin (Cis) for additional 48 h. After treatment, cell viability was measured by SRB assay. The figure shows the combined values of three independent experiments. The columns indicate the mean, and the bars indicate the S.E. (**P<0.01 DOX− *versus* DOX+ *P<0.01 Cis *versus* no Cis). (**b**) ARHI enhanced cytotoxicity to cisplatin in clonogenic assays. SKOv3-ARHI, Hey-ARHI and OVCAR4-ARHI cells were grown in 6-well plates at an initial density of 2000 cells/well and allowed to settle for 24 h, and then cells were treated with DOX, chloroquine and cisplatin as described above in (**a**). Cell viability was measured by clonogenic assays. Data were obtained from three independent experiments. The columns indicate the mean, and the bars indicate the S.E. (**P<0.01, DOX− *versus* DOX+ *P<0.01 Cis *versus* no Cis)

To test whether ARHI-induced autophagy inhibited or enhanced cisplatin-induced cytotoxicity in ovarian cancer cells and whether CQ would modify this effect, viability of SKOv3-ARHI cells and Hey-ARHI cells was determined after induction of autophagy with ARHI and treatment with or without cisplatin and with or without CQ. In preliminary studies, a concentration of CQ was found that functionally inhibited autophagy by blocking LC3 II degradation using western analysis and inhibiting the degradation of autophagic vesicles using immunofluorecent staining (Supplementary Figure S10), but exerted minimal direct cytotoxicity. SKOv3-ARHI or HEY-ARHI cells were pretreated with DOX and/or CQ for 24 h, followed by treatment with cisplatin for an additional 48 h. Viability was measured in short-term cell cultures with SRB (Figure 5a) and SKOv3-ARHI, HEY-ARHI or OVCAR4-ARHI ovarian cancer cell lines. Cell viability was also measured using long-term clonogenic assays (Figure 5b). Cisplatin treatment or ARHI induction with DOX alone significantly reduced cell viability relative to diluent-treated controls (*P*<0.01), whereas cells treated with DOX and cisplatin together resulted in dramatically greater growth inhibition (*P*<0.001) compared with each agent alone (Figures 5a and b), suggesting that ARHI expression and consequent autophagy enhanced the cytotoxic effect of cisplatin. Treatment with CQ did not further enhance cancer cell killing, but rather slightly, however, significantly (*P*<0.05) reduced cytotoxicity in SKOv3-ARHI and OVCAR4-ARHI cells in the absence of cisplatin by long-term clonogenic assay (Figure 5b). Thus, our data indicate that ARHI-induced autophagy in cell culture enhances cisplatin cytotoxicity, rather than protecting tumor cells from apoptosis.

### ARHI re-expression enhances cisplatin-induced apoptosis

**Figure 6:**
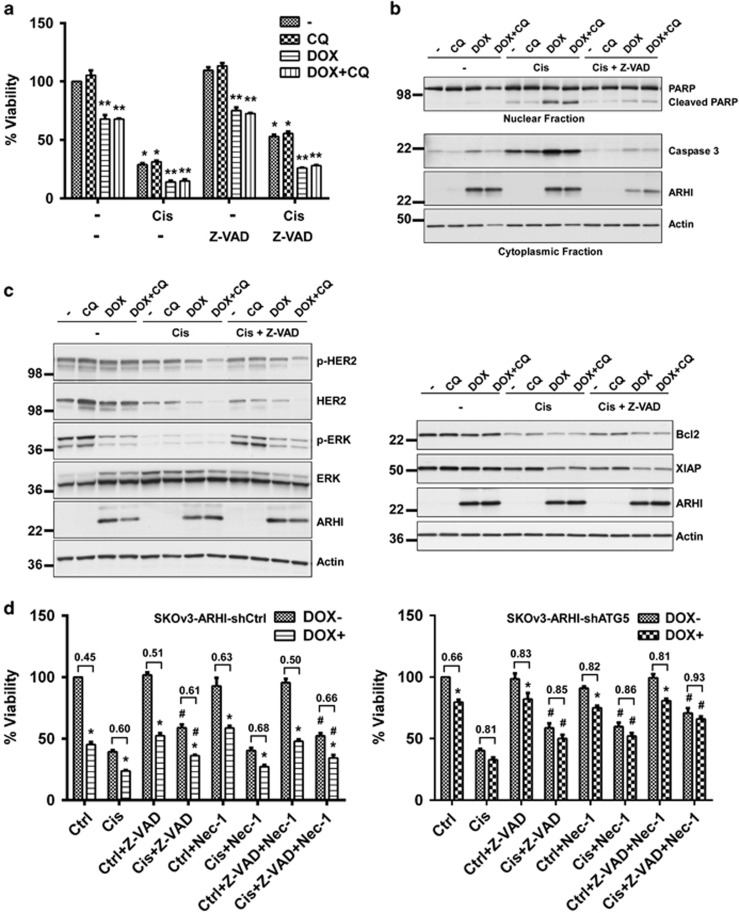
ARHI re-expression enhanced cisplatin-induced apoptosis and ARHI-mediated autophagy-associated cell death enhanced the cytotoxicity of cisplatin in cell culture. (**a**) Cisplatin-induced apoptotic cell death in ARHI-induced autophagic ovarian cancer cells could be partially blocked by Z-VAD in short-term cell cultures. SKOv3-ARHI cells were treated with DOX, chloroquine and cisplatin as described in Figure 5a. Z-VAD (40 *μ*M) and DOX were added simultaneously. Cell viability was measured with SRB assays. Data were obtained from three independent experiments. The columns indicate the mean, and the bars indicate the S.E. (**P<0.01, DOX+ *versus* DOX− *P<0.01 Cis *versus* no Cis). (**b**) Treatment of ARHI-induced autophagic ovarian cancer cells with chloroquine and cisplatin induced activated caspase-3 release and increased PARP cleavage. SKOv3-ARHI cells were pretreated with 5 *μ*M chloroquine and 1 *μ*g/ml DOX for 24 h. Cells were then treated with 5 *μ*M chloroquine, 1 *μ*g/ml DOX and 5 *μ*M cisplatin for 48 h. Cell lysates were collected for western analysis. (**c**) Treatment of ARHI-induced autophagic ovarian cancer cells with chloroquine and cisplatin downregulated ERK and HER2 activity and the expression of XIAP and Bcl-2. Experiments were performed as described in (**b**). (**d**) ARHI-induced growth inhibition was blocked by shATG5 knockdown and partially blocked by Nec-1, but not by Z-VAD. ARHI-enhanced cell death by Cis was blocked by both Z-VAD and Nec-1 in shATG5 knockdown cells. SKOv3-ARHI-shcontrol and SKOv3-ARHI-shATG5 cells were treated with DOX and/or cisplatin as described in Figure 5a. Z-VAD (40 *μ*M) and/or Nec-1 (40 *μ*M) were added with DOX. The figure shows the combined values of three independent experiments. The columns indicate the mean, and the bars indicate the S.E. (*P<0.01, DOX+ *versus* DOX− ^#^*P*<0.01, Cis *versus* Cis+Z-VAD or Cis+Nec-1 or Cis+Z-VAD+Nec-1) and the numbers indicate the ratio of DOX− *versus* DOX+

To determine the mechanism(s) by which ARHI enhanced cisplatin-induced cell death, we first measured the viability of ovarian cancer cells treated with or without cisplatin and with or without Z-VAD, a pan-caspase inhibitor. We found that Z-VAD could partially block cisplatin-induced cell death (Figure 6a), but induction of ARHI still produced additional growth inhibition in the absence (*P*<0.01) or presence (*P*<0.01) of Z-VAD, consistent with the possibility that multiple mechanisms of cell death were associated with the combined treatment (Figure 6a). We next assessed treated cells for the activation of caspase-3 and cleavage of PARP, both classic markers of apoptosis. ARHI induction followed by cisplatin treatment significantly increased caspase-3 activation and PARP cleavage (Figure 6b and Supplementary Figure S11). Increased caspase-3 activation and PARP cleavage could be fully blocked by Z-VAD treatment, and cell viability could not be fully rescued by caspase inhibition alone, again pointing to both apoptotic and non-apoptotic mechanisms of cell death mediated by ARHI either alone or in combination with cisplatin.

### ARHI and cisplatin modulate the expression of Bcl-2 and XIAP by downregulating ERK and HER2 kinase activity

To identify mechanisms by which ARHI expression enhances cisplatin-induced apoptosis, reverse phase protein arrays (RPPAs) were used to monitor changes in signaling during autophagy-associated cell death in the presence and absence of cisplatin. SKOv3-ARHI cells were treated with or without DOX for 24 h and then with cisplatin or diluent for 48 h. Expression of 214 signaling proteins and their phosphorylated derivatives were analyzed by RPPA. Induction of ARHI followed by treatment with cisplatin downregulated ERK and HER2 phosphorylation. To validate the RPPA results, positive hits were confirmed by western blot analysis. Cisplatin and ARHI strongly inhibited ERK and HER2 phosphorylation and decreased the expression of Bcl-2 and XIAP (X-linked inhibitor of apoptosis) proteins (Figure 6c). Thus, cisplatin and ARHI induced the occurrence of apoptosis that results from decreasing ERK and HER2 activation and downregulation of their downstream targets, Bcl-2 and XIAP, the known inhibitors of apoptosis.

### Autophagy is required for ARHI enhancement of cisplatin cytotoxicity

Having demonstrated that ARHI induces autophagy-mediated necroptosis, we next asked whether autophagy could also modulate cisplatin-induced apoptosis. SKOv3-ARHI-shATG5 and SKOv3-ARHI-shcontrol cells were treated with DOX for 24 h before treatment with cisplatin for an additional 48 h. As previously documented, treatment with cisplatin or induction of ARHI inhibited SKOv3-shControl cell growth and a greater effect was observed with a combination of cisplatin treatment and ARHI induction. Z-VAD partially blocked cisplatin and ARHI-induced cell death and Nec-1 could only partially prevent ARHI-induced cell death in SKOv3-ARHI-shcontrol cells (Figure 6d). Combined treatment with Z-VAD and Nec-1 did not further inhibit cell death in autophagic cells treated with cisplatin. ATG5 knockdown significantly rescued ARHI-mediated loss of cell viability. Inhibition of apoptosis and necroptosis with a combination of Z-VAD and Nec-1 further blocked ARHI- and cisplatin-induced cell death in shATG5 knockdown cells (Figure 6d). Western analysis demonstrated that cells with ATG5 knockdown exhibited less conversion of LC3 I to LC3 II and formed fewer mature autophagosomes after DOX-induced ARHI re-expression compared with shControl cells treated with DOX. ATG5 knockdown partially blocked the cisplatin- and ARHI-induced reduction of ERK and HER2 phosphorylation and Bcl-2 and XIAP expression (Supplementary Figure S12). Together, our data suggest that autophagy and necroptosis contribute to cell death observed after ARHI re-expression and cisplatin treatment, as well as to the cell signaling events that enhance cisplatin-induced apoptosis.

### ARHI expression enhances chemosensitivity to cisplatin in ovarian cancer xenografts

**Figure 7:**
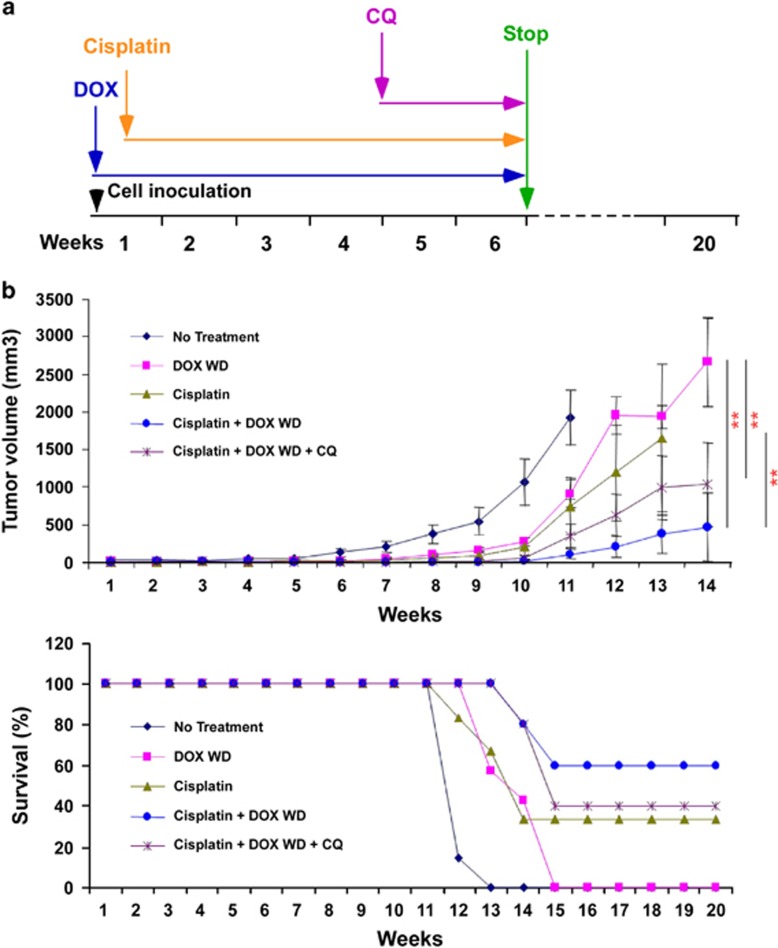
ARHI re-expression enhanced chemosensitivity to cisplatin *in vivo.* (**a**) A diagram of treatment for murine xenografts performed in (**b**). (**b**) ARHI and cisplatin inhibited the outgrowth of dormant autophagic ovarian cancer cells in ovarian cancer xenografts. Balb/c nu/nu mice were injected s.c. with 5 × 10^6^ SKOv3-ARHI cells and given water with or without DOX for 6 weeks. They were injected i.p. as indicated with cisplatin once per week for the first 6 weeks, and with chloroquine 5 days per week for weeks 5 and 6. Tumor diameters were measured once per week and volumes calculated. Kaplan–Meier survival curves were calculated for the control group and treatment groups. Tumor growth curve and survival curves were plotted with Microsoft Excel. The bars indicate the S.E. (**P<0.01, DOXWD *versus* cisplatin+DOXWD; DOXWD *versus* cisplatin+DOXWD+CQ)

In cell culture, induction of ARHI at physiologic levels in SKOv3 cells decreased clonogenic growth by 90% within 3 days.^16^ When human SKOv3-ARHI ovarian cancer cells were grown as xenografts in immunosuppressed mice, induction of ARHI significantly inhibited xenograft growth, but dormant cells survived. When ARHI induction was withdrawn, xenografts grew promptly to kill the host.^16^ Treatment of mice bearing dormant xenografts with CQ dramatically delayed xenograft growth following withdrawal of ARHI.^16^ To find whether dormant autophagic cells were still susceptible to treatment with cisplatin and whether additive antitumor effects could be seen with the addition of CQ, subcutaneous xenografts were established using SKOv3-ARHI cells. ARHI expression was induced by providing DOX in drinking water immediately after injection of cancer cells. Controls received water without DOX. Weekly treatment with cisplatin began on day 3 after cancer cell injection, whereas daily CQ injection began on day 28 and DOX was withdrawn on day 42 (Figure 7a). As in our previous study,^16^ induction of dormancy and autophagy with DOX significantly inhibited tumor growth and prolonged survival (*P*<0.05; Figure 7b), but all tumors grew progressively following removal of DOX at 42 days. In the absence of ARHI induction with DOX, weekly treatment of xenograft tumors with cisplatin also decreased tumor growth and prolonged survival (*P*<0.01; Figure 7b). Induction of ARHI with DOX and weekly treatment cisplatin had a greater effect than either alone (*P*<0.05; Figure 7b and Supplementary Figure S13). Addition of CQ did not further inhibit dormant xenograft growth. Consequently, dormant ovarian cancer cells were still susceptible to treatment with cisplatin and ARHI expression enhanced the cytotoxic effect of cisplatin. Functional inhibition of autophagy delayed outgrowth of cancer cells, but did not affect cisplatin-induced inhibition of dormant ovarian cancer growth.

## Discussion

The mechanism(s) of autophagy-associated cell death are not well understood. Here we document for the first time that ARHI-induced autophagy enhances production of ROS, leading to RIP1/RIP3-mediated necroptosis. Expression of ARHI is downregulated in ovarian cancers by multiple mechanisms.^17, 18, 32, 33^ Re-expression of ARHI drives autophagy by interacting at several points in the development of autophagolysosomes.^15, 16, 20^ Persistent re-expression of ARHI produces loss of clonogenic potential of ovarian cancer cells within 3 days in cell culture.^16^ Autophagy-associated ARHI-induced cell death is associated with modest G1 cell-cycle arrest and necrosis, but neither apoptosis nor senescence. ARHI-induced cell killing and increased levels of ROS depend critically on autophagy. Knockdown of ATG5 decreases ROS and rescues ovarian cancer cells from necrotic death after ARHI re-expression. Necroptosis, one form of necrosis, appears to be a mechanism of ARHI-induced cell death. RIP1 kinase activity is crucial for necroptosis induced by Fas, TNF and TRAIL death receptors,^34^ and Nec-1, an allosteric inhibitor of RIP1 kinase, abolishes necroptosis-specific RIP3 phosphorylation and inhibits death receptor-induced necroptosis in different cellular models.^35^ In our study, Nec-1 partially rescued ovarian cancer cells from ARHI-mediated cell death by 40–50% (Figure 4d). After ARHI re-expression, RIP1 coprecipitated not only with RIP3, but also with ARHI and LC3. ARHI and LC3 both associate with the membrane of autophagosomes and can be chemically crosslinked.^16^ Thus, ARHI may facilitate the formation of autophagosome–necrosome co-complexes by binding directly or indirectly with RIP1 and RIP3 proteins inducing necroptosis.

The level of ARHI expression is also likely to be important in determining the mechanism of cell death. In this report, ARHI has been re-expressed at physiologic levels,^16^ and an earlier study from our group found that overexpression of ARHI by infection of SKOv3 ovarian cancer cells and MDA-MB-231 breast cancer cells with ARHI adenovirus induced apoptosis through a caspase-independent, calpain-dependent mechanism.^36^ Recently, Li *et al.*^37^ also reported that overexpression of ARHI in TOV12D and ES2 ovarian cancer cells induced apoptosis and autophagic cell death by inhibiting activity of AKT and decreasing the expression of Bcl-2.

Autophagy can enhance or inhibit the response of cancer cells to chemotherapy, depending on the particular experimental model. With SKOv3-ARHI and Hey-ARHI ovarian cancer cell lines we have found that ARHI re-expression enhances chemosensitivity to cisplatin in cell culture. ARHI re-expression increases cisplatin-induced apoptosis that depends upon autophagy and the downregulation of Bcl-2 and XIAP. Aberrant overexpression of Bcl-2 and XIAP is associated with tumorigenesis and increased resistance to chemotherapy in multiple malignancies.^38, 39^ Bcl-2 is a potent anti-apoptotic protein that protects cells from diverse stress challenges.^40^ A key function of Bcl-2 is to act as guardian of mitochondrial integrity by opposing pro-apoptotic Bcl-2 family members, and XIAP is also a potent inhibitor of caspases and apoptosis. XIAP can directly bind to and inhibit both the initiator and effector caspases and inhibit both mitochondria-dependent and mitochondrial-independent apoptotic pathways.^41, 42^ Treatment of ovarian cancer cells with cisplatin downregulated Bcl-2 and XIAP. Re-expression of ARHI further increased the cisplatin-mediated suppression of Bcl-2 and XIAP expression and enhanced the accumulation of activated caspase-3 and cleaved PARP. In previous studies, our group and others have found that re-expression of ARHI inhibits both Ras-MAP and PI3K signaling.^15, 16, 43^

*In vivo*, ARHI re-expression enhanced chemosensitivity to cisplatin in SKOv3 ovarian cancer xenografts. In studies with cells in culture, the addition of CQ, a functional inhibitor of autophagy, slightly decreased the combined cytotoxic effect of ARHI and cisplatin on cells. Thus, it appears that in this system ARHI re-expression and consequent autophagy enhances, rather than inhibits, cisplatin-reduced cytotoxicity. In our previous report,^16^ induction of ARHI significantly inhibited xenograft growth when human SKOv3-ARHI ovarian cancer cells were grown as xenografts in mice, and dormant cells survived. When ARHI induction was withdrawn, xenografts grew promptly to kill the host.^16^ Treatment of mice bearing dormant xenografts with CQ dramatically delayed xenograft growth following withdrawal of ARHI.^16^ In the present study, cisplatin delayed the outgrowth of xenografts and the addition of CQ did not modify cisplatin activity, although a modest effect might be lost because of the group size. Previously, various reports also revealed that autophagy can function as a pro-survival pathway in the metabolically stressed tumor microenvironment,^44, 45^ suggesting that autophagy can function in a highly context-dependent manner as a pro-survival or pro-death mechanism.

To date, our studies have been conducted with ovarian cancer where ARHI is downregulated in 60% of primary cancers, but expressed in 80–90% of residual, potentially dormant nodules of residual cancer found at second-look operations.^20^ Residual ARHI-positive cancer cells exhibit punctate staining for MAP-LC3 consistent with autophagy. These observations are likely to prove relevant to several other cancers in which ARHI is downregulated, including breast, lung, prostate, pancreatic, hepatic and thyroid cancers.^46, 47, 48, 49^

## Materials and Methods

### Antibodies and reagents

Antibodies against *β*-Actin, Bcl2, p-ERK, ERK, LC3, p62, XIAP, ATG5 and PARP were purchased from Cell Signaling Technology (Danvers, MA, USA). Anti-XIAP and anti-pP27 were purchased from BD Biosciences (San Jose, CA, USA). Anti-Caspase-3 (Active) antibody was purchased from Millipore (Temecula, CA, USA). An anti-ARHI murine monoclonal antibody (ID8) was generated in our laboratory. Doxycycline hyclate (DOX), chloroquine diphosphate salt (CQ), cis-diammine-platinum (II) dichloride (Cisplatin), Z-VAD, Nec-1 and *N*-acetyl-L-cysteine were purchased from Sigma-Aldrich (St. Louis, MO, USA).

### Cell culture

Tet-on inducible SKOv3-ARHI ovarian cancer cells (TP53 null and mutations in PIK3CA and ARID1A) were grown in McCoy's medium supplemented with 10% FBS, 200 *μ*g/ml G418 and 0.12 *μ*g/ml puromycin.^16^ Tet-on inducible Hey-ARHI ovarian cancer cells (TP53 wild type and mutations in KRAS and BRAF) were cultured in RPMI-1640 medium supplemented with 10% FBS, 25 *μ*g/ml blasticidin and 1 *μ*g/ml puromycin. Stable ATG5 knockdown and nontargeted control cell lines were generated by transducing SKOv3-ARHI or Hey-ARHI ovarian cancer cells with lentivirus encoding each shRNA (shATG5 Fisher V3LHS_301131; shControl Fisher RHS4348, Pittsburgh, PA, USA). Cells were propagated in medium, and GFP-positive cells were sorted by flow cytometry before the experiments. ATG5 protein levels were examined by western immunoblotting to measure the degree of knockdown in each experiment.

### SRB cell proliferation assays

Cells (2000 per well) were grown in 96-well plates in 100 *μ*l media per well. The cells were cultured overnight, and then treated with or without DOX (to induce ARHI) and with or without CQ (to block autophagic flux) for 24 h. Medium was changed and cells incubated with or without cisplatin and with or without DOX for 48 h. For experiments with an apoptosis inhibitor (Z-VAD) and/or a necroptosis inhibitor (Nec-1), inhibitors were added to cells at the same time as cisplatin, and then cells were incubated for 48 h. For experiments with siRIP1 and siRIP3 knockdown, cells were reversely transfected with siRIP1 (50 *μ*M) or siRIP3 (50 *μ*M) or siRIP1 plus siRIP3 (25+25 *μ*M), and then 24 h after siRNA transfection, cells were treated with or without DOX for additional 48 h. Cell viability was assessed with SRB assay. Briefly, 50 *μ*l 30% TCA was added to each well and plates were incubated at 4 °C for 1 h. The plates were rinsed with distilled H_2_O and 100 *μ*l of 0.4% SRB in 1% acetic acid was added to each well. Plates were incubated for 30 min at room temperature, and then rinsed with 1% acetic acid. SRB was solubilized with 100 *μ*l of 10 mM Tris buffer for 5 min with shaking. Absorbance values were measured on a microplate reader at 570 nm.

### Clonogenic assays

Cells were plated in 6-well plates at a density of 2000 cells per well and cultured overnight. Cells were then incubated for 24 h with or without DOX (to induce ARHI) and with or without CQ treatment (to block autophagic flux). Medium was then changed and cells incubated with or without cisplatin and with or without DOX for 48 h. After treatment, cells were grown for an additional 12 days. Colonies were stained with Coomassie blue and counted.

### Hoechst 33342 (BF) and PI staining

SKOv3-ARHI, SKOv3-ARHI-shCtrl and SKOv3-ARHI-shATG5 cells were treated with DOX for different intervals. Live cells were incubated with Hoechst 33342 (BF, Cell Signaling, Danvers, MA, USA) at a final concentration of 2 *μ*g/ml for 15 min at room temperature, and PI was added (Sigma-Aldrich) and slides were incubated for an additional 5 min in a final concentration of 0.625 *μ*g/ml. Images were obtained with immunofluorescence microscopy. Necrotic cells were identified by staining with both PI and BF dyes. More than 200 cells were counted for each sample.

### Flow cytometry

The percentage of cells in different phase of the cell cycle was determined based on relative DNA content as determined by flow cytometry analysis. SKOv3-ARHI and Hey-ARHI cells were treated with DOX at indicated times to induce ARHI expression. Then, cells were detached by incubating with 0.05% trypsin-EDTA, washed with PBS and fixed overnight in 70% ethanol. Fixed cells were then centrifuged, washed, resuspended in PBS containing RNase A and PI (50 *μ*g/ml each) and incubated for 20 min at 37 °C with gentle shaking. Stained cells were filtered through nylon mesh (41-*μ*m pore size) and analyzed on a Coulter flow cytometer XL-MCL (Coulter Corporation, Miami, FL, USA). The percentages of sub-G1 population and cell-cycle distribution were determined using the MULTICYCLE software program (Phoenix Flow Systems, San Diego, CA, USA).

### Immunoprecipitation and immunoblotting

SKOv3-DIRAS3 cells were incubated in lysis buffer (50 mM Hepes, pH 7.0, 150 mM NaCl, 1.5 mM MgCl_2_, 1 mM EGTA, 10 mM NaF, 10 mM sodium pyrophosphate, 10% glycerol, 1% Triton X-100) plus protease and phosphatase inhibitors (1 mM PMSF, 10 *μ*g/ml leupeptin, 10 *μ*g/ml aprotinin and 1 mM Na_3_VO_4_). Cells were lysed for 30 min on ice, and then centrifuged at 17 000 × *g* for 30 min at 4 °C. The protein concentration was assessed using a bicinchoninic acid (BCA) protein assay (Thermo, Waltham, MA, USA). Lysates (0.8–1 mg protein) were diluted with lysis buffer to 1 ml. Immune complexes were incubated overnight with 2 *μ*g of the antibody and precipitated with protein G magnetic beads (Thermo Scientific, Pittsburgh, PA, USA) for 60 min. Complexes were washed in lysis buffer (3 × 5 min) and in PBS (3 × 5 min). Immunoprecipitated proteins were separated by SDS-PAGE and transferred to PVDF membranes. Immunoblot analysis was performed with the indicated antibodies and visualized with an ECL (enhanced chemiluminescence) detection kit (GE Healthcare, Pittsburgh, PA, USA).

### ApopTag fluorescein and ARHI immunofluorescence costaining

Apoptag Fluorescein *in situ* Apoptosis Detection kit was from EMD Millipore (Billerica, MA, USA). Cells seeded on coverslips were fixed in 1% formaldehyde in PBS for 5 min, rinsed twice in PBS and then permeabilized in ethanol/acetic acid 2 : 1 for 5 min at −20 °C. Coverslips were rinsed twice in PBS and equilibration buffer was added immediately for 10 s at room temperature followed by incubation with anti-ARHI antibody and TdT enzyme at 37 °C for 1 h. Cells were incubated twice with stop/wash buffer for 10 min in PBS and incubated with antidigoxigenin conjugate and secondary antibody (Alexa Fluor 594, Grand Island, NY, USA) for 30 min at room temperature. Coverslips were then washed four times for 5 min each in PBS and mounted on glass slides with Vectashield Fluorescent Mounting medium with DAPI (Vector Labs, Burlingame, CA, USA). Images were obtained with immunofluorescence microscopy.

### Senescence (SA-*β*-gal) assays

SKOv3-ARHI and Hey-ARHI cells were grown in 6-well plates at an initial density of 20 000 cells per well. After 48 h of incubation with DOX to induce ARHI expression, cells were washed with PBS and fixed in 4% paraformaldehyde before staining with X-gal solution according to the manufacturer's instructions (Senescence Cell Histochemical Staining kit, Sigma-Aldrich). After cells were incubated in the staining solution for 16 h at 37 °C, *β*-galactosidase-positive cells with blue precipitate were counted using bright-field microscopy.

### ROS measurement

The total free radical levels in cultured cells and supernatant were measured by The OxiSelect *in vitro* ROS Assay Kit (Cell Biolabs) according to the manufacturer's instructions. Briefly, all samples were added to assay wells with a catalyst that promoted acceleration of the oxidation reaction. After 15 min of incubation, dichlorodihydrofluorescein was added to each well to quantitate the oxidation reaction. Fluorescence intensity was measured and ROS calculated relative to a hydrogen peroxide standard. In order to block free radical scavengers, *N*-Acetyl-L-cysteine was added to all wells in the 96-culture plates at a final concentration of 1 mM.

### Murine xenografts

Six-week-old BALB/c nu/nu mice were purchased from MD Anderson Cancer Center Department of Veterinary Medicine and Surgery (Houston, TX, USA). SKOv3-ARHI cells (5 × 10^7^) were injected subcutaneously into the flank of each mouse. DOX (2 mg/ml) in 5% sucrose or sucrose alone was added to the drinking water on the day of injection. On the following day, mice were injected i.p. with 2 *μ*g/g cisplatin once per week for 6 weeks. During the fourth and fifth weeks, mice were injected with 50 *μ*g/g CQ 5 days per week. DOX was withdrawn from the drinking water after 6 weeks. Tumors were measured once per week using a digital caliper. All procedures were carried out according to the animal protocol approved by the institutional animal care and use committee of the M.D. Anderson Cancer Center at the University of Texas.

### Statistical analysis

All experiments were repeated independently at least twice and the data expressed as mean±S.E. Statistical analysis was performed using Student's *t-*test (two-sample assuming unequal variances). Differences were considered statistically significant at *P*<0.05 (two sided).

## Abbreviations

CQ: chloroquine
DIRAS3: GTP-binding RAS-like 3
DOX: doxycycline
Nec-1: necrostatin-1
ROS: reactive oxygen species
RPPA: reverse phase protein array
NAC: *N*-acetyl cysteine
