# Supplementary material for: ARHI (DIRAS3)-mediated autophagy-associated cell death enhances chemosensitivity to cisplatin in ovarian cancer cell lines and xenografts
Source: Cell Death Dis. 2015 Aug 6;6(8):e1836–. doi: 10.1038/cddis.2015.208 (PMC4558501; doi:10.1038/cddis.2015.208)
Supplement: Supplementary Information [file cddis2015208x1.doc]

Supplemental information

**ARHI (DIRAS3)-mediated autophagy associated cell death enhances chemosensitivity to cisplatin in ovarian cancer cell lines and xenografts**

Michele N. Washington*, Grace Suh*, Aaron F. Orozco*, Yan Wang, Weiqun Mao, Margie N. Sutton, Hailing Yang, Steven Millward, Argentina Ornelas, Neely Atkinson, Warren Liao, Robert C. Bast, Jr** and , Zhen Lu**

Department of Experimental Therapeutics, University of Texas M.D. Anderson Cancer Center, Houston, TX 77030-4009, USA

**^*^** Authors contributed equally to this work

**^**^**Correspondence:

The University of Texas M. D. Anderson Cancer Center, Unit 1439

1400 Pressler Street, Houston, TX 77030

Tel.: 713-792-7743, Fax: 713-742-7864

Email: [rbast@mdanderson.org](mailto:rbast@mdanderson.org)

Email: [zlu@mdanderson.org](mailto:zlu@mdanderson.org)


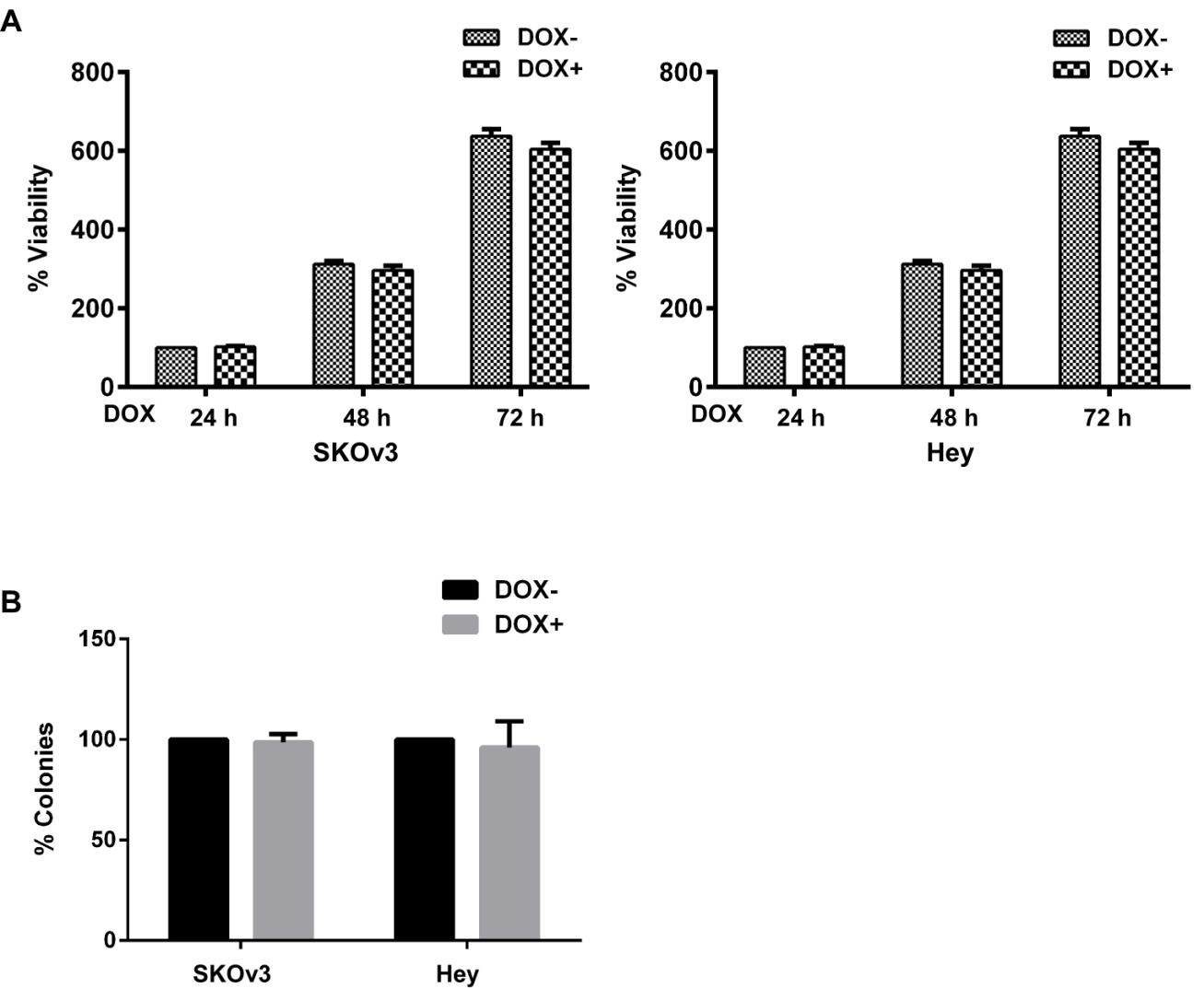


**Figure. S1.**  **Doxycycline did not produce cell growth arrest and clonogenic cell death in parental SKOv3 and Hey cells.** (**A**) Doxycycline did not inhibit cell growth in short-term cell culture. SKOv3-ARHI cells and Hey-ARHI cells were treated with 1 ug/ml DOX (to induce DIRAS3) for indicated time. Cell viability was measured with SRB analysis. The figure shows the combined values of 3 independent experiments. The columns indicate the mean, and the bars indicate the S.E. (**B**) Doxycycline did not inhibit clonogenic cell growth. Cells were plated in 6-well plates, at a density of 2000 cells/well and allowed to settle for 24 h. Cells were then treated with 1 ug/ml DOX for 3 days and incubated for up to 14 days. Cell viability was measured by colony counts. Data were obtained from three independent experiments. The columns indicate the mean, and the bars indicate the S.E.

**
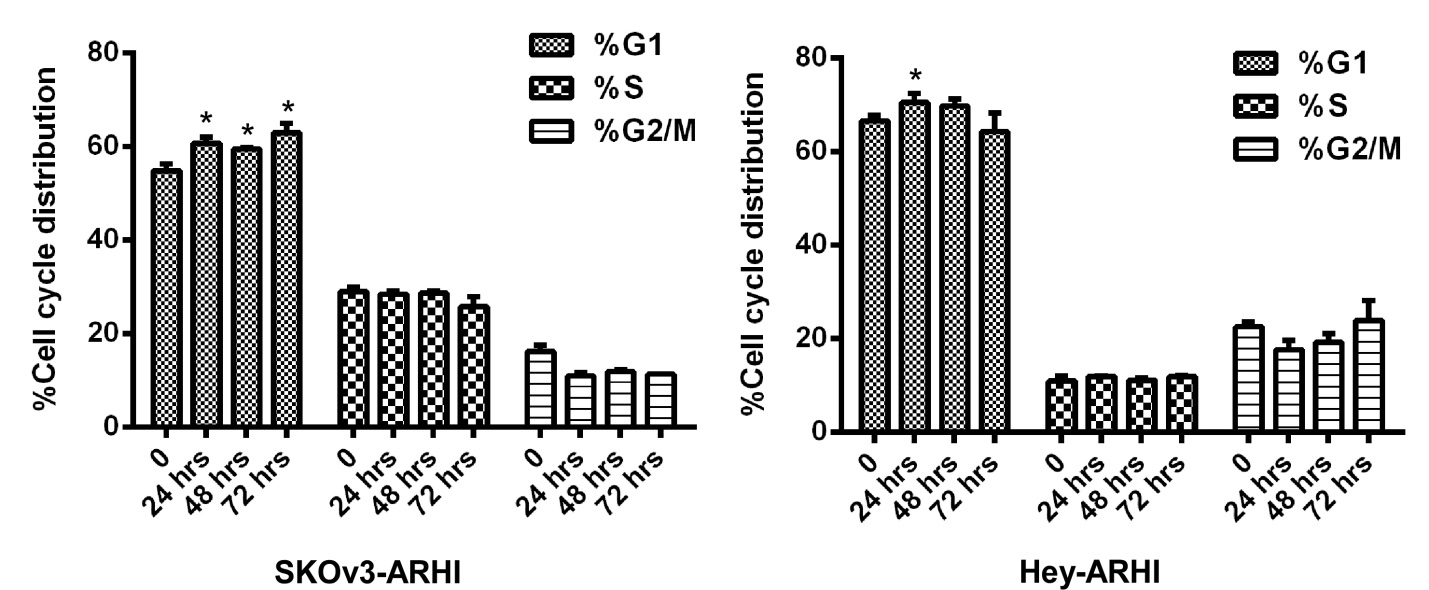
**

**Figure. S2. ARHI expression induces G1 cell cycle arrest.** SKOv3-ARHI and Hey-ARHI cells were treated with DOX as indicated time to induce ARHI expression. The percentage of cells in different phase of the cell cycle was determined based on relative DNA content as measured by flow cytometry analysis. Data were obtained from three independent experiments. The columns indicate the mean, and the bars indicate the S.E. (* *p*<0.05).


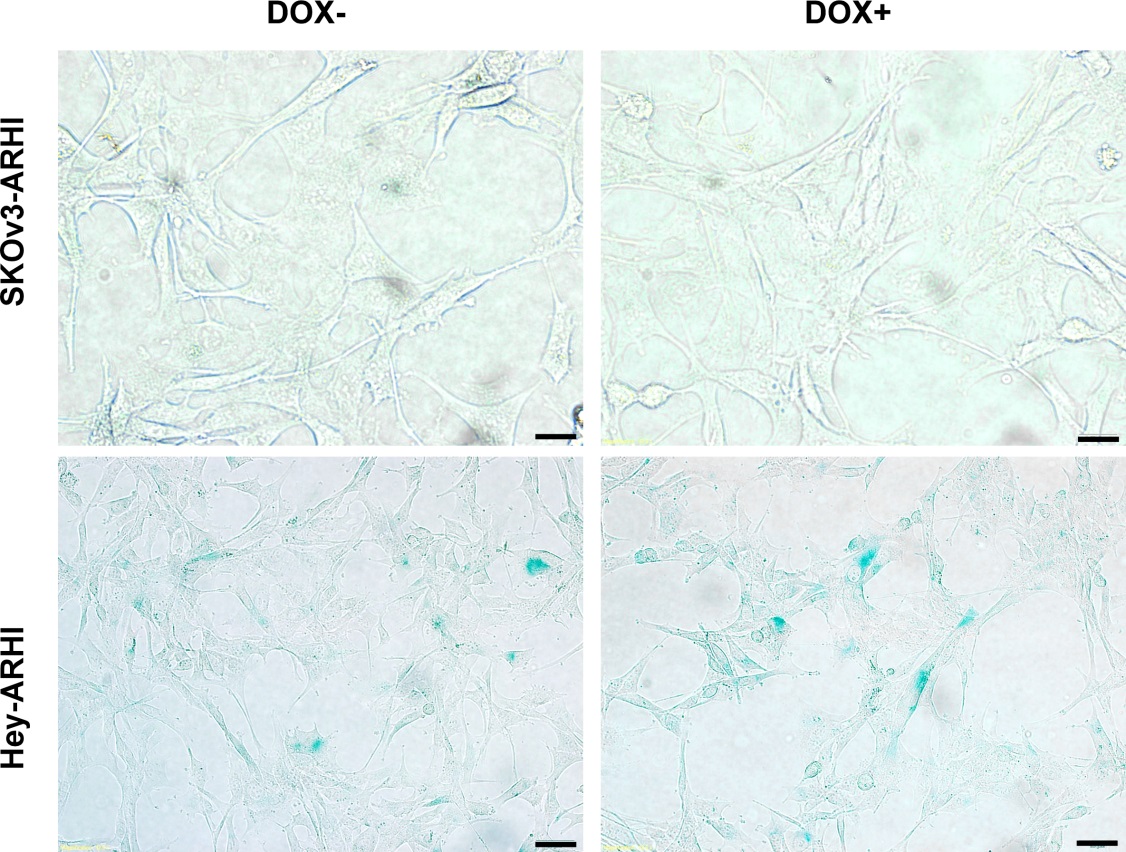


**Figure. S3. ARHI expression failed to induce senacesence** SKOv3-ARHI and Hey-ARHI cells were examined by β-galactosidase positive cells in absent or present of DOX treatment (for inducing ARHI expression). The positive cells were determined by blue precipitate using bright field microscopy. Scale bars: 10 µm.

**
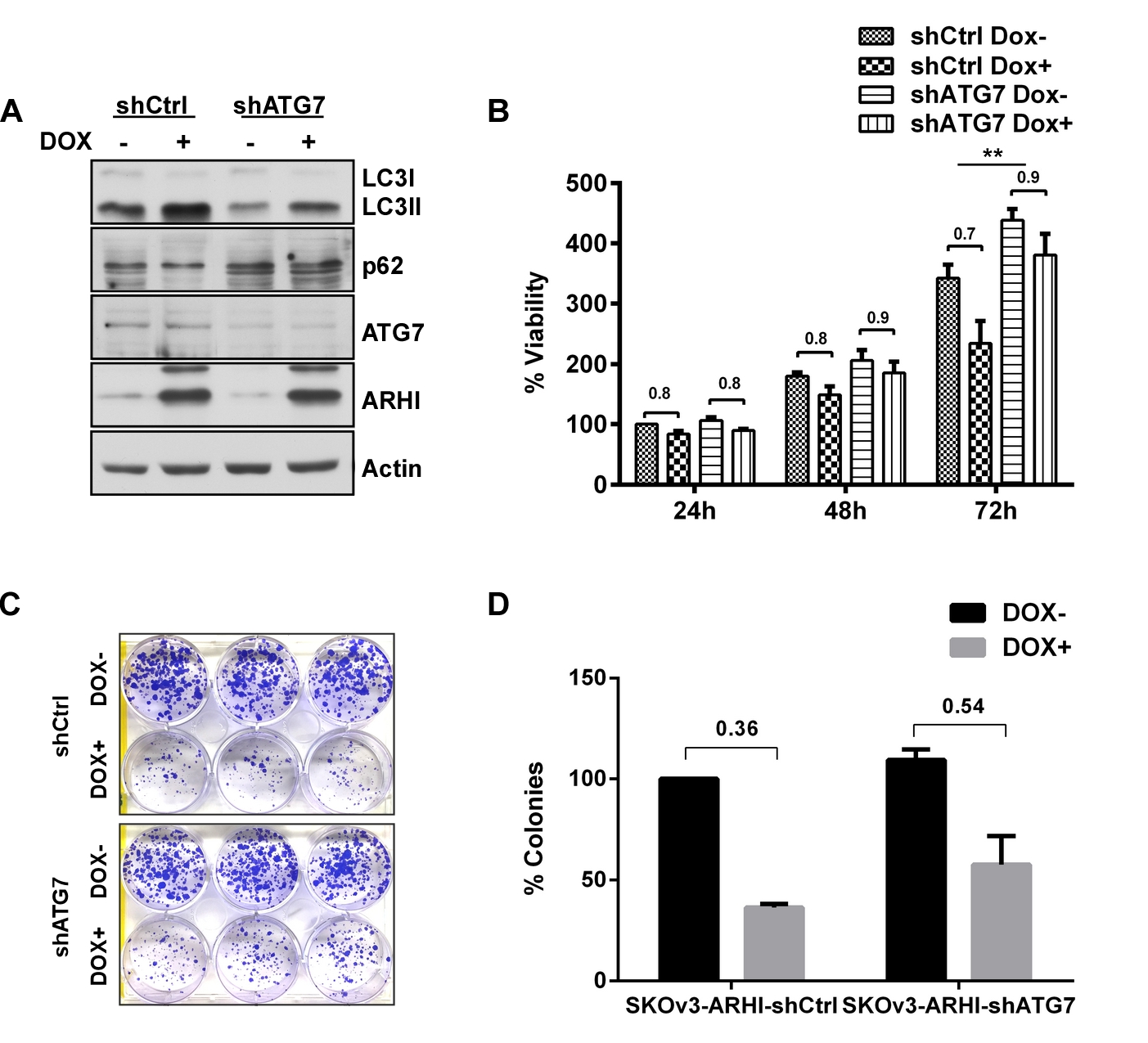
**

**Figure S4.** **ARHI re-expression induced autophagic cell death.** (**A**) ATG7 knockdown blocked ARHI-induced autophagy. SKOv3-ARHI-shControl/shATG7 cells were treated with DOX for 24hr, then cell lysates were collected and probed with antibodies against LC3, p62, ATG7, DIRAS3 and Actin. (**B-F**) ATG7 knockdown blocked ARHI-induced cell death. SKOv3-ARHI-shControl/shATG7 cell viability was measured with SRB assays (**B**) and clonogenic assays (**C-D**) as done in Figure 1A. The columns indicate the mean, and the bars indicate the S.E. The numbers indicate the ratio of shCtrl DOX- vs shCtrl DOX+ and ratio of shATG7 DOX- vs shATG7 DOX+. Differences of ratio between shCtrl and shATG7 were considered statistically significant at *p*<0.01 (**).

**
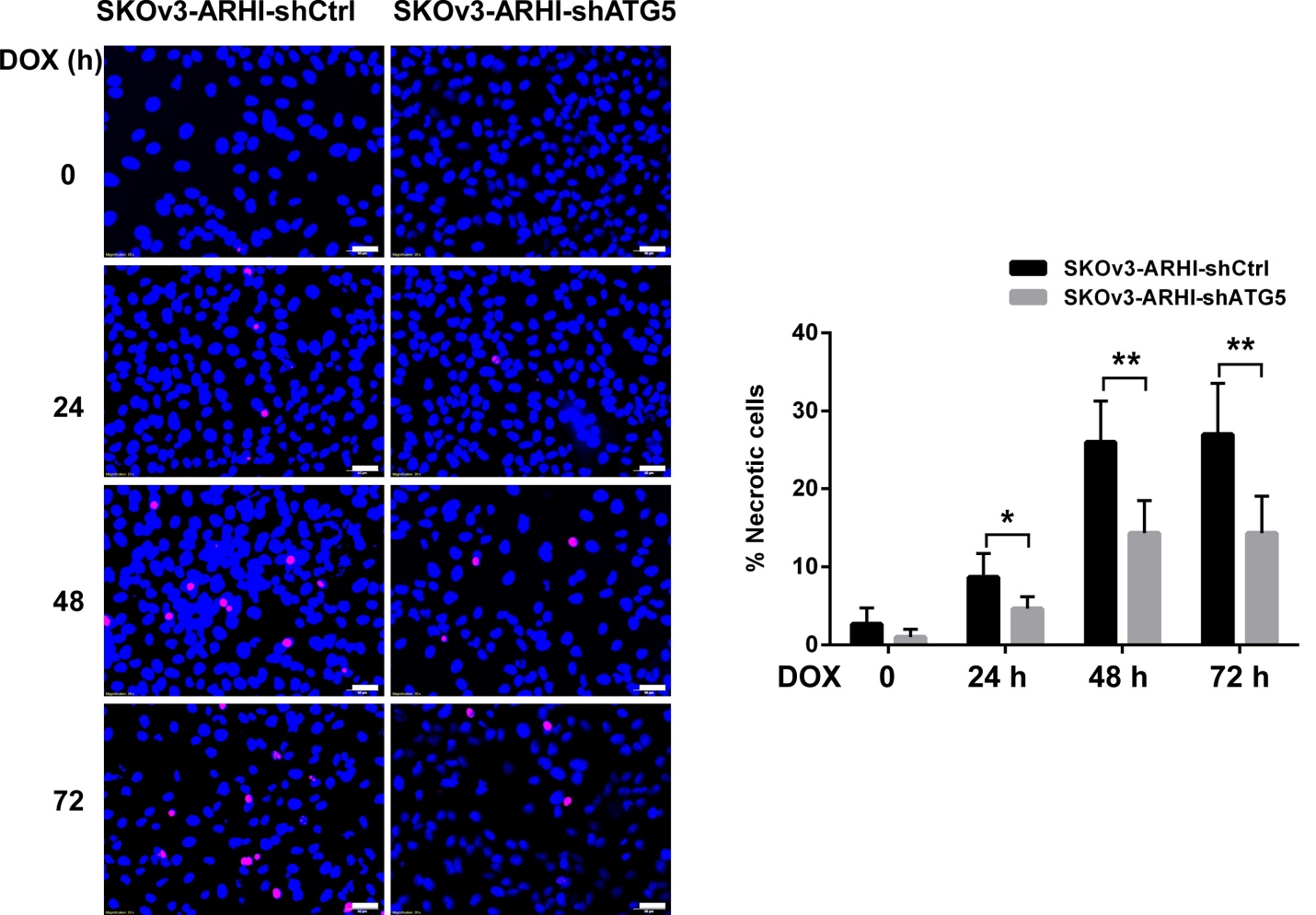
**

**Figure. S5. Knock down ATG5 decreased ARHI-induced necrosis.** SKOv3-ARHI-shCtrl and SKOv3-ARHI-shATG5 cells were treated DOX as indicated times. Live cells were stained with Hoechst 33342 and PI dye at final concentration of 2 µg/mL and 0.625 µg/mL respectively. The necrosis cells were identified based on the positive staining with PI and Hoechst dye. More than 200 cells were counted for each sample. Data were obtained from three independent experiments. The columns indicate the mean, and the bars indicate the S.E. (* *p*<0.05; ***p*<0.01). Scale bars: 50 µm.


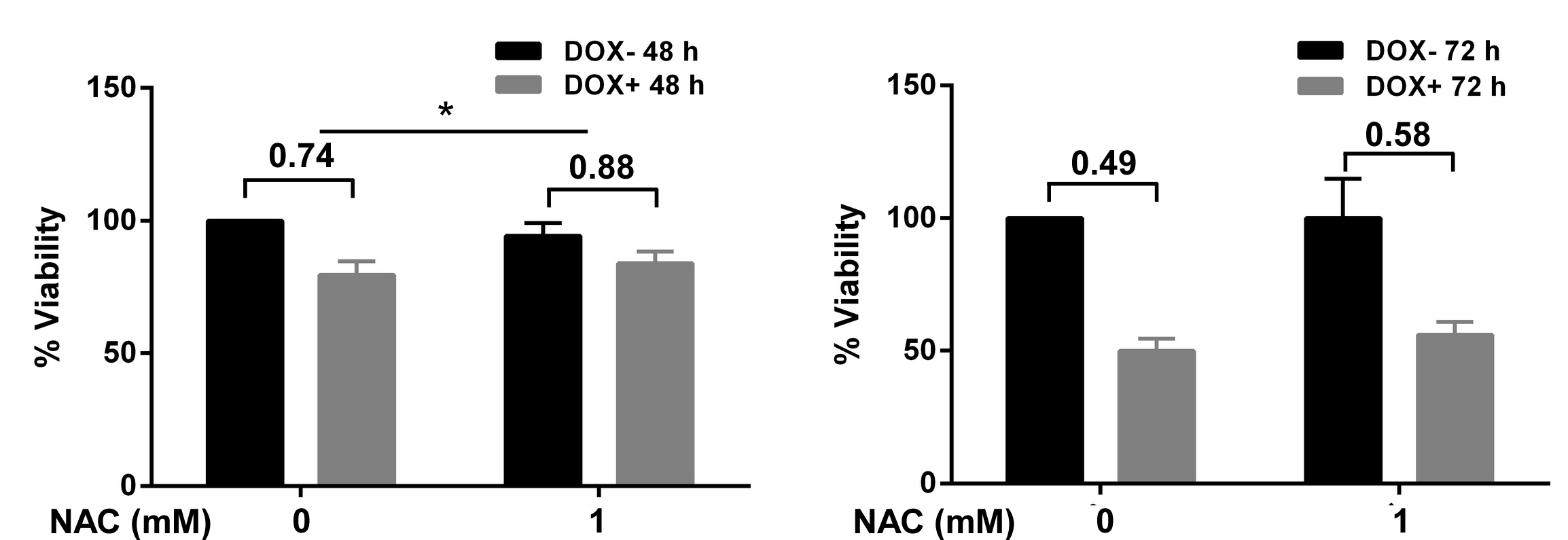


**Figure S6.** ***N*-Acetyl-L-cysteine (a free radical scavenger) significantly rescued ARHI-induced loss of cell viability.** SKOv3-ARHI cells were treated with DOX and *N*-Acetyl-L-cysteine (1mM) simultaneously as indicated times. Cell viability assay was measured with SRB assay. The figure shows the combined values of three independent experiments. The columns indicate the mean, and the bars indicate the S.E. The numbers indicate the ratio of DOX- vs DOX+. Differences between NAC treated and untreated were considered statistically significant at *p*<0.05 (*).


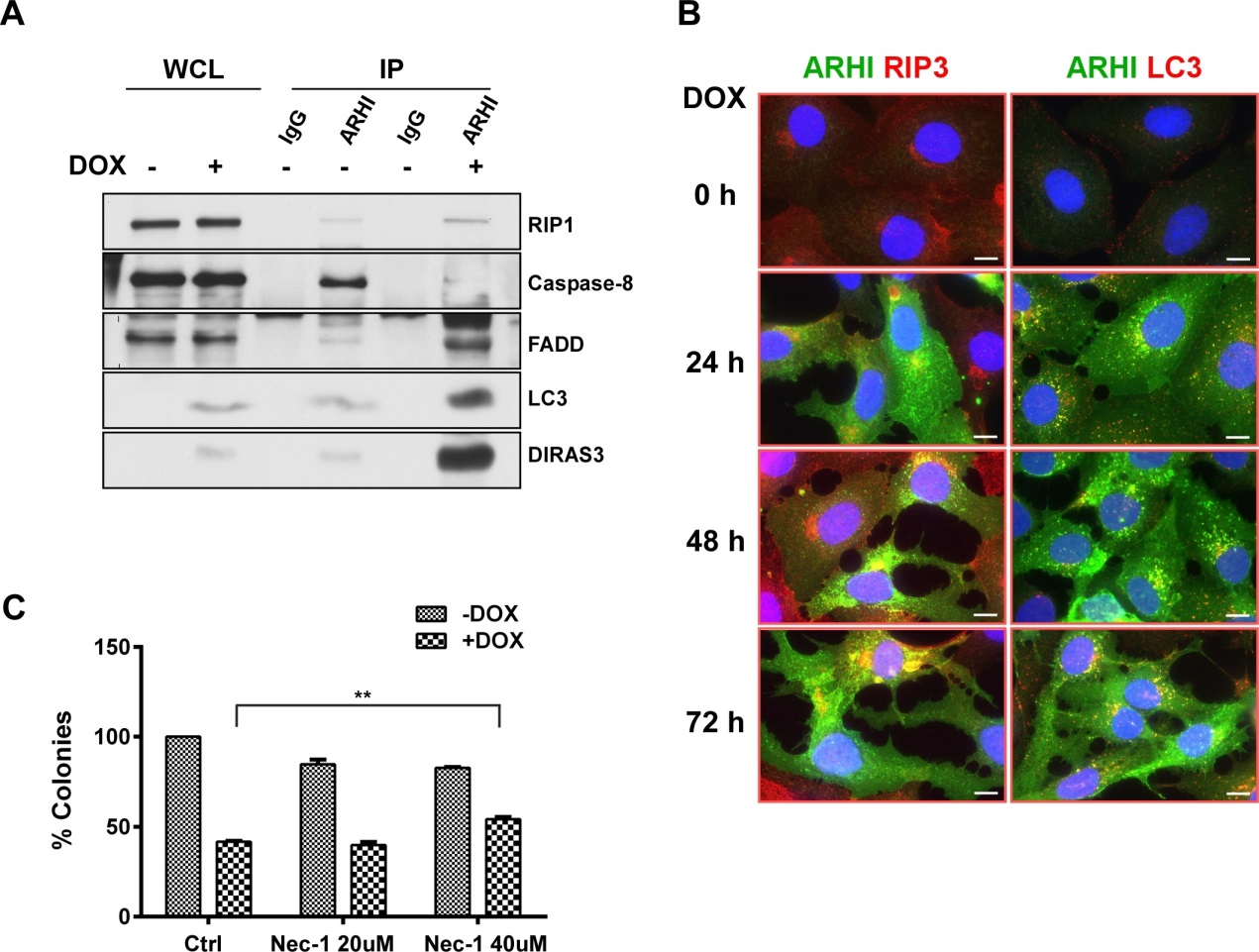


**Figure S7. ARHI interacted with RIP1 and LC3 and Necrotatin-1 (Nec-1) decreased ARHI-induced clonogenic growth inhibition.** (**A**) DIRAS3 co-immunoprecopitated with RIP1 and LC3. SKOv3-DIRAS3 cells were treated with or without DOX. Endogenous RIP1/FADD/Caspase8/ARHI/LC3 complexes were immunoprecipitated with anti-ARHI antibody and analyzed for co-immunoprecipitation of RIP1/FADD/Caspase8/ARHI/LC3 conjugates (IP). Host species-matched nonspecific IgG served as negative controls. Whole-cell lysates (WCL) are included for comparison. (**B**) DIRAS3 co-localizes with LC3 and RIP3. SKOv3-DIRAS3 cells were treated with DOX to induce ARHI expression for the times indicated. Immunofluorescence staining of DIRAS3, endogenous LC3 and RIP3 were analyzed by confocal microscopy. Scale bars: 10 μm. (**C**) Nec-1 partially rescued clonogenic cell growth. Cells were plated in 6-well plates, at a density of 2000 cells/well and allowed to settle for 24 h. Cells were then treated with 1 ug/ml DOX for and Nec-1 simultaneously for 3 days, and then incubated for up to 14 days. Cell viability was measured by colony counts. Data were obtained from three independent experiments. The columns indicate the mean, and the bars indicate the S.E. (***p*<0.01).


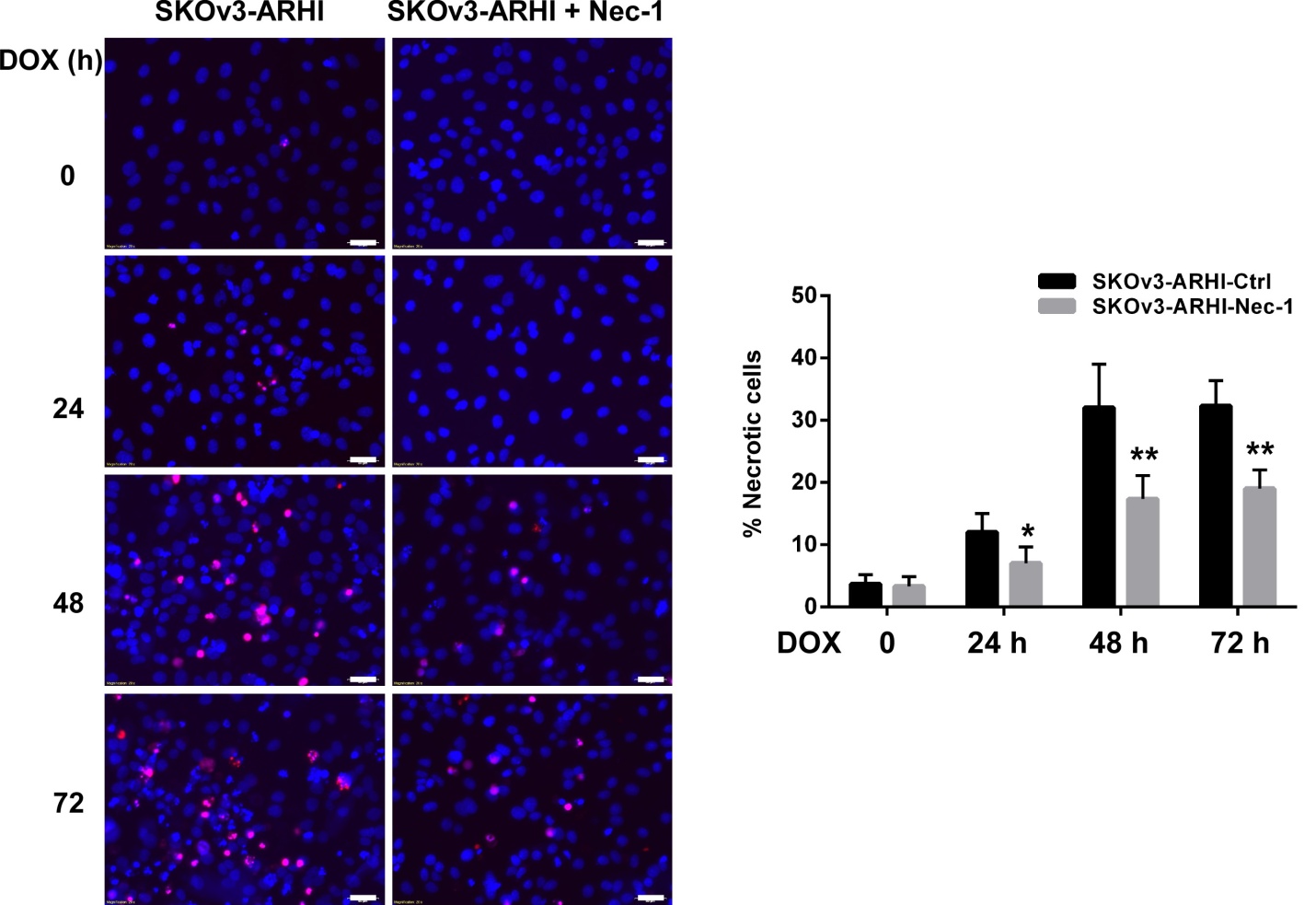


**Figure S8. Necrotatin-1 (Nec-1) decreased ARHI-induced necrosis**. SKOv3-ARHI cells were treated with DOX and Nec-1 (40uM) simultaneously as indicated time. Live cells were stained with Hoechst 33342 and PI dye at final concentration of 2 µg/mL and 0.625 µg/mL respectively. The necrosis cells were identified based on the positive staining with PI and Hoechst dye. More than 200 cells were counted for each sample. Data were obtained from three independent experiments. The columns indicate the mean, and the bars indicate the S.E. (* *p*<0.05; ***p*<0.01). Scale bars: 50 µm.

**
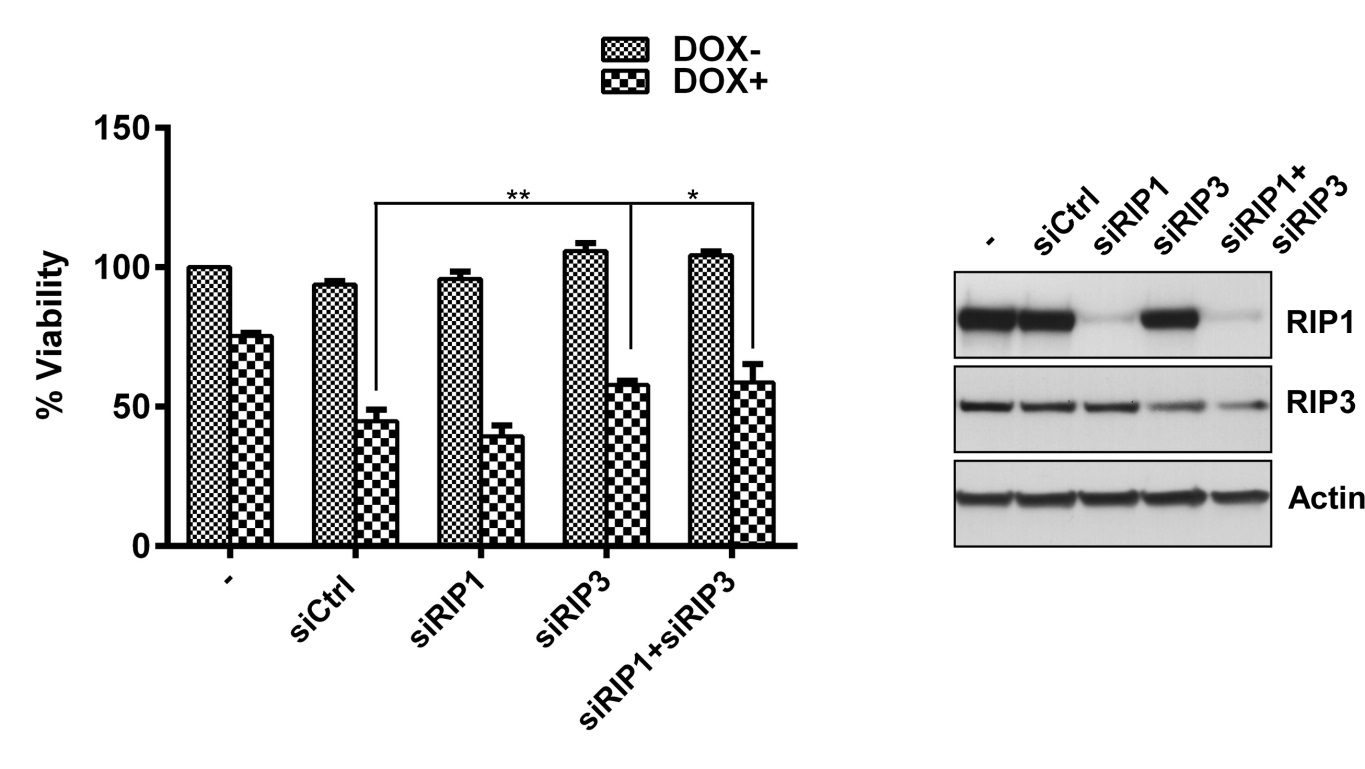
**

**Figure S9. RIP1 and RIP3 knockdown decreased ARHI-mediated growth inhibition.** SKOV3-ARHI cells were reversely transfected with siRIP1 (50 µM) or siRIP3 (50 µM) or siRIP1 plus siRIP3 (25 µM + 25 µM), and then 24 h after siRNA transfection, cells were treated with or without DOX for additional 48 h. Cell viability was measured with SRB assay. Knockdown efficiency of siRNA was examined by western analysis. Data were obtained from two independent experiments. The columns indicate the mean, and the bars indicate the S.E. (* p<0.05; **p<0.01).


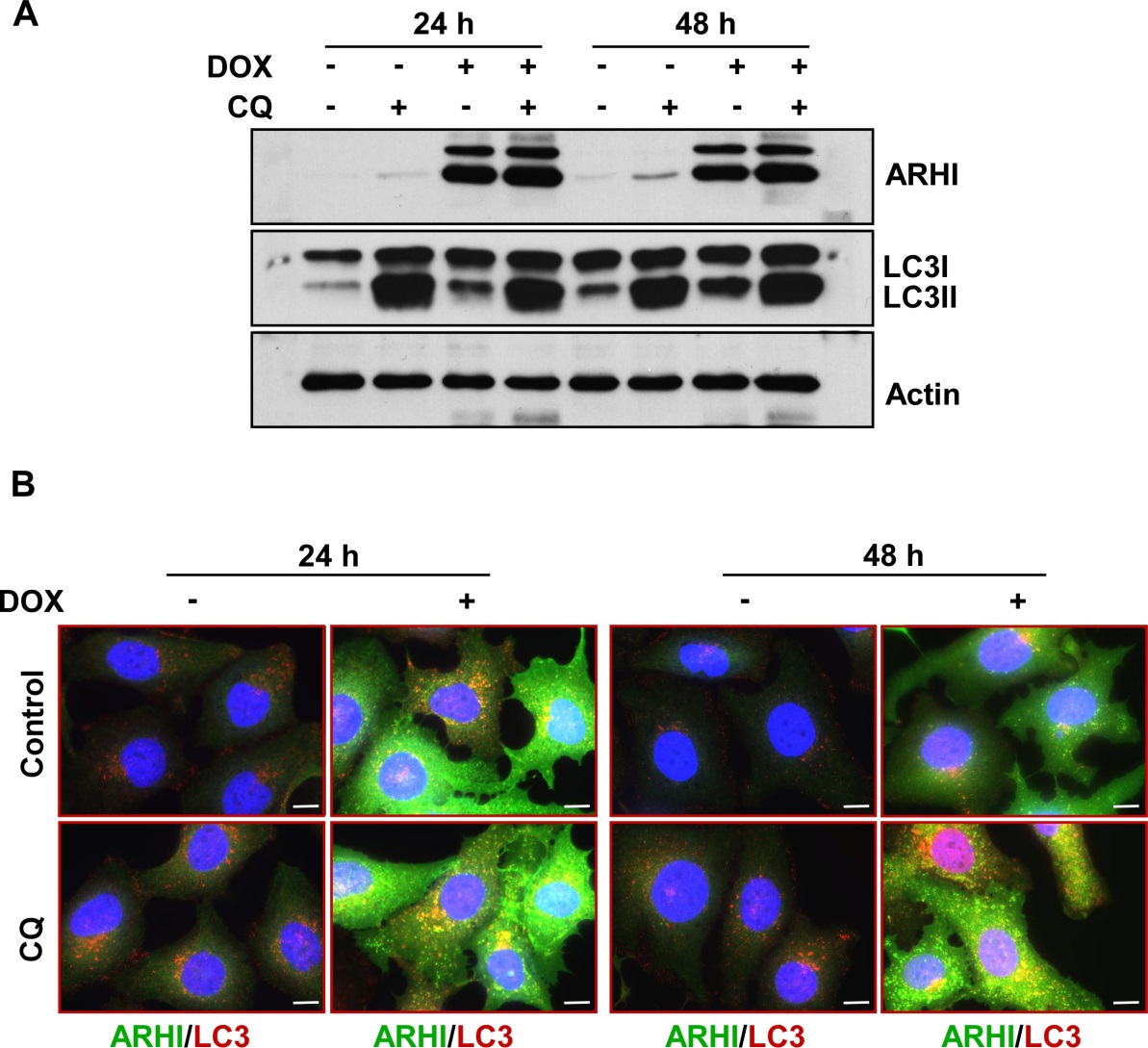


**Figure S10**. **Chloroquine (CQ) blocked the degradation of autophagic vesicles.** (A) Chloroquine treatment increased LC3II by blocking LCII degradation. SKOv3-DIRAS3 cells were treated with or without DOX and CQ for indicated time, and then cell lysates were collected and probed with antibodies against LC3, DIRAS3 and Actin with western analysis. (B) Chloroquine treatment increased autophagosomes by blocking the degradation of autophagic vesicles. SKOv3-DIRAS3 cells were treated with or without DOX and CQ for indicated time, and then Immunofluorescence staining of DIRAS3 and endogenous LC3 were analyzed by confocal microscopy. Scale bars: 10 μm.


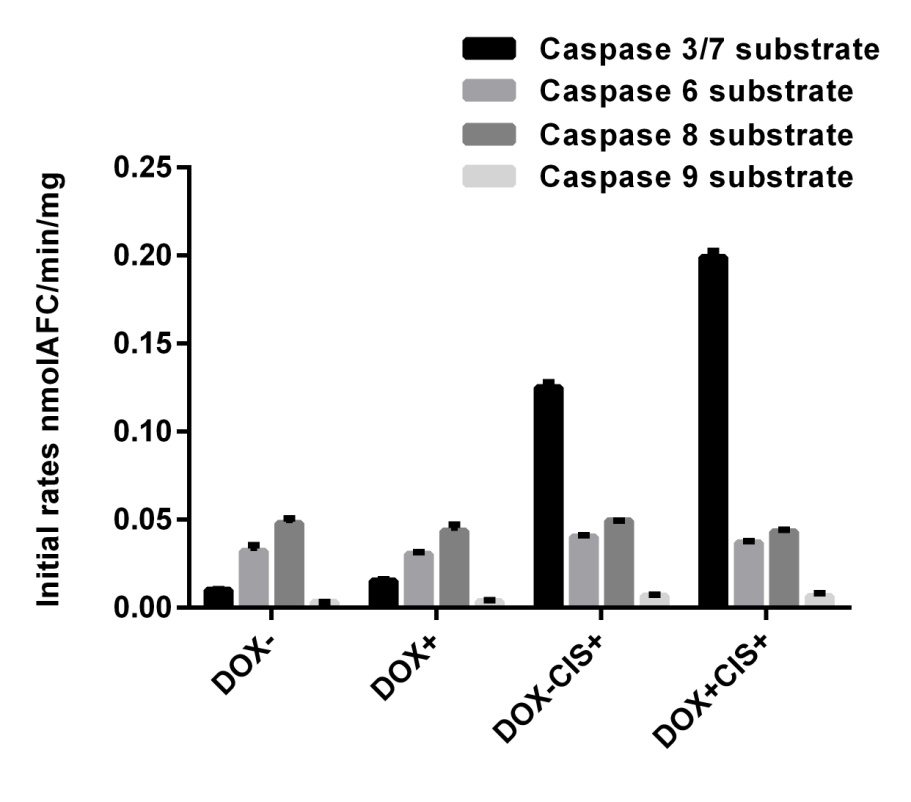


**Figure S11. ARHI enhanced cisplatin-mediated caspase activity in SKOv3-ARHI Cells.** SKOv3-ARHI cells were treated with or without doxycycline (DOX) for 72 h and with or without cisplatin (CIS) for 48 h. Cell lysates were incubated with a panel of fluorescent caspase substrates. 6.7 mM of each substrate was incubated with 20 mL of cell lysate and monitored at lex = 400 nm, lem = 505 nm for 90 minutes using a Biotek Synergy plate reader. The caspase substrates were as follows: Casp3/7 -AcDEVD-AFC, Casp6 - AcVEID-AFC, Casp8 - AcIETD-AFC, Casp9 - AcLEHD-AFC. Initial rates were calculated in GraphPad and normalized to total protein concentration of the lysate.


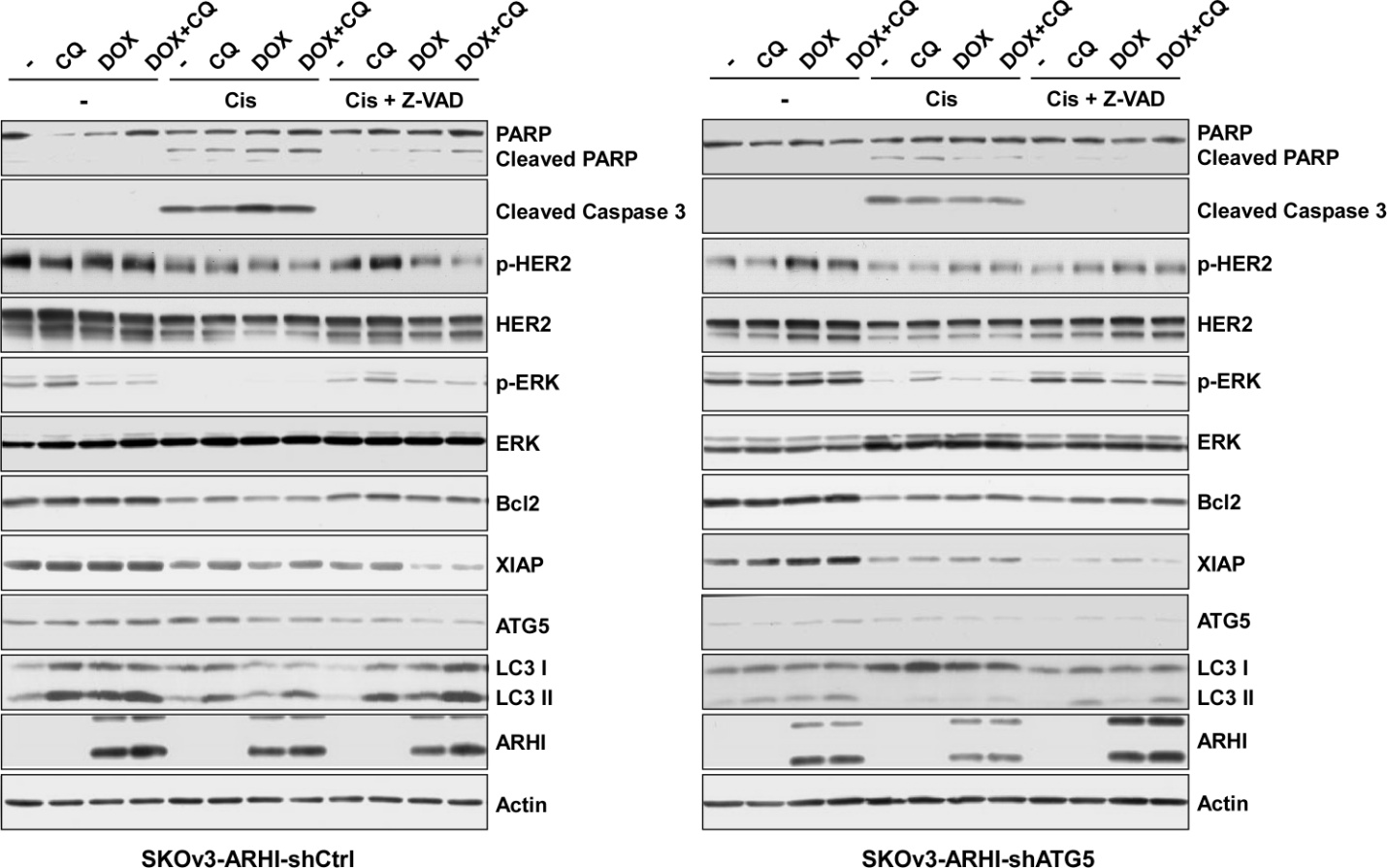


**Figure S12. ARHI-mediated autophagy associated cell death enhanced the cytotoxicity of cisplatin in cell culture.** SKOv3-ARHI cells were pre-treated with 5 uM chloroquine and 1 ug/ml DOX for 24 h. Cells were then treated with 5 uM chloroquine (CQ), 1 ug/ml DOX, and 5 uM cisplatin (Cis) for additional 48 h. Cell lysates were obtained for Western blot analysis. Experiments were repeated three times.


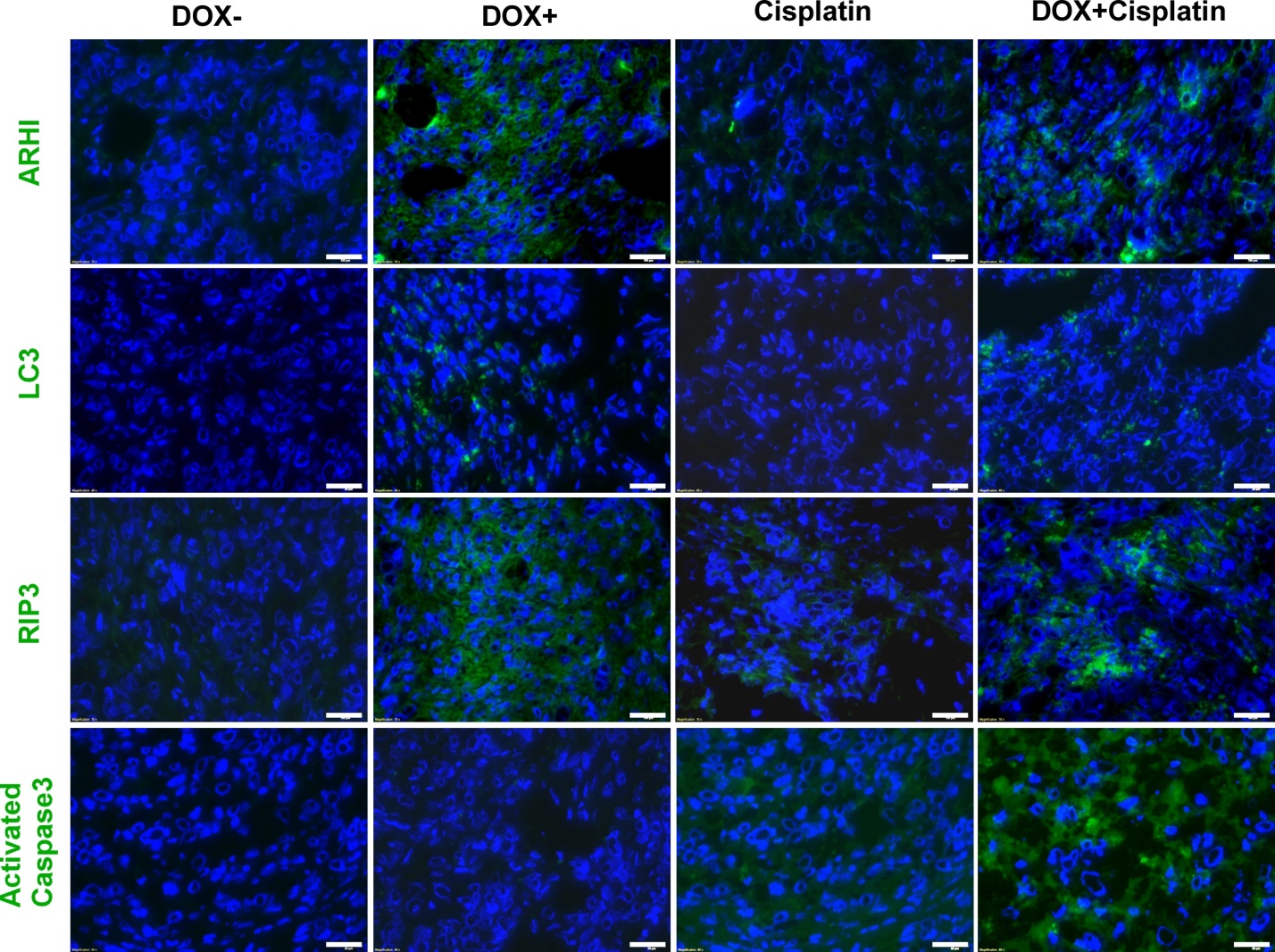


**Figure S13**. **ARHI induced autophagy-mediated necroptosis and cisplatin induced apoptosis in ovarian cancer xenografts**. Tumor Xenografts (from Figure 7 experiment) were excised and then embedded in OTC. Immunofluorescence staining of DIRAS3, endogenous LC3, RIP3 and activated caspase3 were analyzed by confocal microscopy. Scale bars: 20 μm.
